# Comprehensive genetic interaction analysis of the *Bacillus subtilis* envelope using double-CRISPRi

**DOI:** 10.1016/j.cels.2025.101406

**Published:** 2025-10-03

**Authors:** Byoung-Mo Koo, Horia Todor, Jiawei Sun, Jordi van Gestel, John S. Hawkins, Cameron C. Hearne, Amy B. Banta, Kerwyn Casey Huang, Jason M. Peters, Carol A. Gross

**Affiliations:** 1 Department of Microbiology and Immunology, University of California, San Francisco, San Francisco, CA 94158, USA; 2 Department of Bioengineering, Stanford University, Stanford, CA 94305, USA; 3 Pharmaceutical Sciences Division, School of Pharmacy, University of Wisconsin-Madison, Madison, WI 53705, USA; 4 Department of Microbiology and Immunology, Stanford University School of Medicine, Stanford, CA 94305, USA; 5 Chan Zuckerberg Biohub, San Francisco, CA 94158, USA; 6 Department of Cell and Tissue Biology, University of California, San Francisco, San Francisco, CA 94158, USA; 7 California Institute of Quantitative Biology, University of California, San Francisco, San Francisco, CA 94158, USA; 8 These authors contributed equally; 9 Lead contact

## Abstract

Understanding bacterial gene function remains a major challenge. Double-mutant genetic interaction analysis addresses this challenge by uncovering the functional partners of targeted genes, enabling association of genes of unknown function with known pathways and unraveling of connections among well-studied pathways, but such approaches are difficult to implement at the genome scale. Here, we use double-CRISPR interference (CRISPRi) to systematically quantify genetic interactions at scale for the *Bacillus subtilis* cell envelope, including essential genes. We discover >1,000 genetic interactions, some known and others novel. Our analysis pipeline and experimental follow-ups reveal the shared and distinct roles of paralogous genes such as *mreB* and *mbl* in peptidoglycan and teichoic acid synthesis and identify additional genes involved in the well-studied process of cell division. Overall, our study provides valuable insights into gene function and demonstrates the utility of double-CRISPRi for high-throughput dissection of bacterial gene networks, providing a blueprint for future studies in diverse species. A record of this paper’s transparent peer review process is included in the supplemental information.

## In brief

Koo et al. apply genome-scale double-CRISPRi to map cell envelope gene interactions in *Bacillus subtilis*, revealing >1,000 genetic interactions and uncovering gene networks in envelope biogenesis and cell division. This study establishes double-CRISPRi as a powerful tool for dissecting bacterial gene networks.

## Graphical Abstract

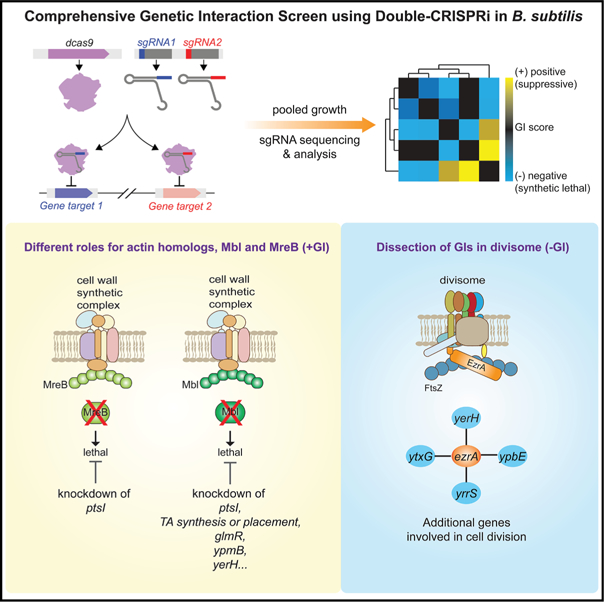

## INTRODUCTION

The field of genetics has been built on deducing gene functions by associating mutations with phenotypes. The ability to investigate the phenotypes of gene disruption mutants in bacteria in multiple conditions at the genomic scale using high-throughput techniques such as transposon insertion libraries,^1,2^ single-gene deletion collections,^3,4^ and CRISPR interference (CRISPRi) libraries^5–8^ has dramatically advanced the pace of discovery of gene functions and enabled unbiased discovery of functional partners through shared phenotypes. Such chemical-genomic studies have predicted functions for thousands of previously uncharacterized or poorly characterized genes and revealed unknown connections between cellular pathways. Nonetheless, studies with hundreds of distinct conditions have failed to identify any phenotypes for a significant fraction of genes (∼30% or more) in even the best-studied model organisms, such as *Escherichia coli*.^4^ Determining the functions of these genes, many of which are broadly conserved, is an outstanding problem.

Genetic interaction (GI) mapping, a cornerstone of classical genetic approaches,^9^ compares the phenotypes of double-deletion mutant strains to the sum of their single-knockout phenotypes. Differences from the null expectation are indicative of GIs, thereby revealing the interacting partners of a gene product and uncovering phenotypes for members of partially redundant gene pairs. The power of this approach has been demonstrated by numerous studies that mapped the GIs between a single gene and the rest of the genome (1 × all) to discover novel protein functions like undecaprenyl flippases,^10^ peptidoglycan (PG) polymerase regulators,^11^ and PG hydrolase co-factors.^12^ Despite the utility of double-mutant analyses, genome-scale GI screens have thus far been executed only in the yeast *Saccharomyces cerevisiae*,^13^ for which automated construction and analysis of >20 million double mutants revealed overall construction principles of the cell. The bottleneck to general use of large-scale GI analysis is that screening requires constructing double mutants in large pools and then determining the identity of both affected genes, even when they are distant from each other on the chromosome. These challenges can be overcome using CRISPRi. Two genes can be transcriptionally repressed by adjacently encoded single-guide RNAs (sgRNAs), and the sgRNAs can be identified and enumerated via sequencing. The utility of a CRISPRi-based GI approach was demonstrated by interrogating 222,784 double knockdown (KD) strains (472 × 472 genes) in mammalian cells,^14^ but such an approach has not yet been reported in bacteria at the genomic scale.

Here, we develop double-CRISPRi technology in the model gram-positive bacterium *Bacillus subtilis* and use it to perform a genome-scale GI screen of cell envelope-related genes. We chose to focus on the gram-positive cell envelope, which is composed of the inner membrane (IM), the PG cell wall, and associated molecules such as teichoic acids (TAs),^15^ for several reasons. First, the envelope is responsible for cellular integrity, elongation, and division, and for mediating environmental, pathogenic, and symbiotic interactions. Second, since the envelope is the target of many antibiotics,^16–18^ the identification of synthetic-lethal gene pairs can aid the design of synergistic antibiotic therapies. Third, envelope processes are difficult to reconstitute biochemically, as they have numerous components and often function across multiple length scales,^19,20^ making genetic dissection paramount. However, the partial redundancy of envelope-function genes, which is necessary to ensure robust growth across conditions, has complicated genetic dissection.^21–23^ Finally, despite intense study over decades, the cell envelope still contains the highest fraction of proteins of unknown function.^24,25^

Our experiments identified >1,000 positive and negative GIs. By combining our screen with follow-up experiments, including live cell microscopy, we uncover links between diverse envelope processes that expand our understanding of the gram-positive cell envelope and provide a valuable resource and discovery tool for the research community. Our study also provides a natural stepping-stone toward the eventual goal of full genome-wide screens, which remain technically and financially challenging due to their size (∼4,000 × ∼4,000 genes = ∼16 million total strains).

## RESULTS AND DISCUSSION

### Construction of a chromosomally encoded double-CRISPRi library

**Figure 1.**
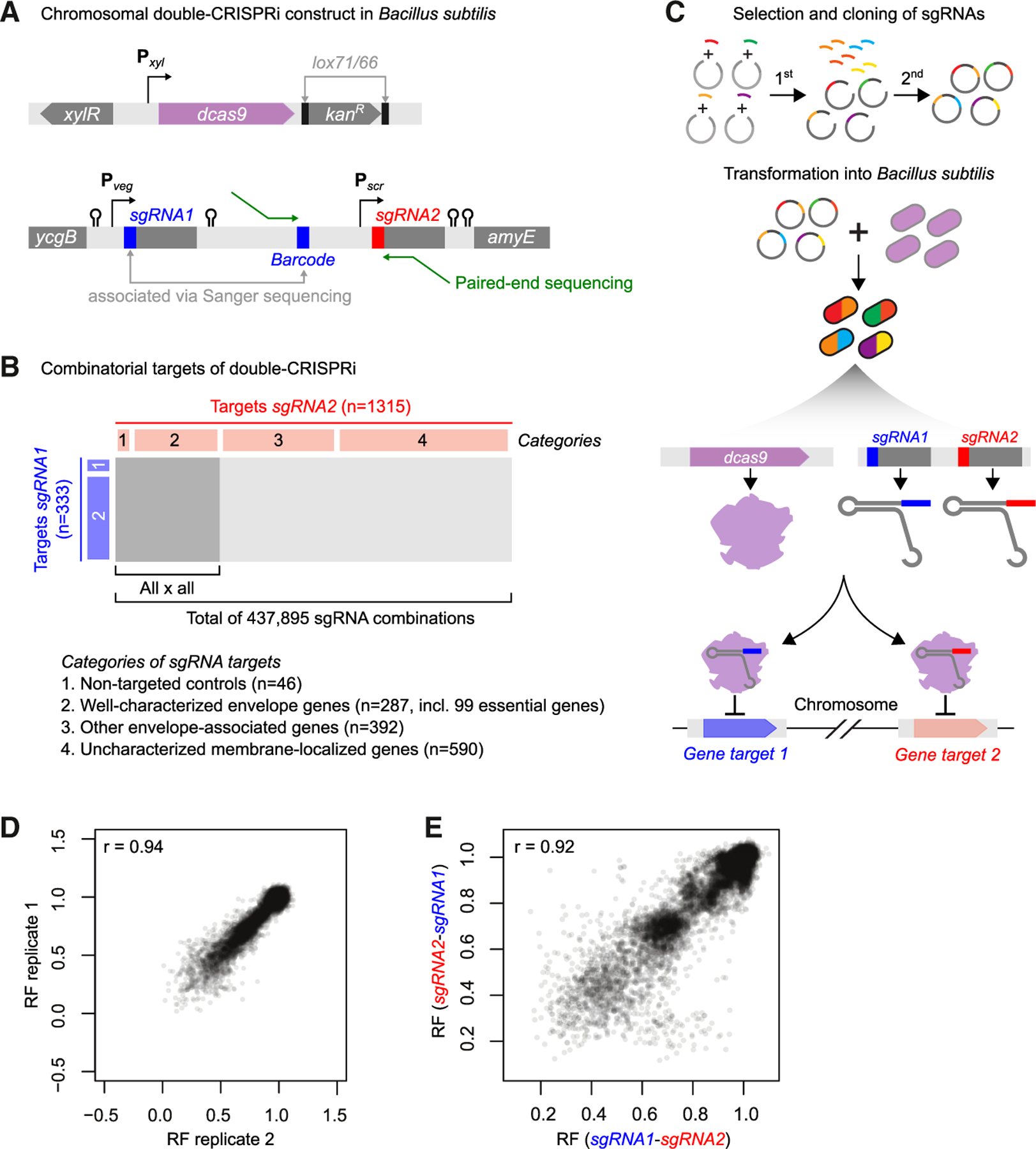
A large-scale inducible, chromosomally integrated double-CRISPRi system in *B. subtilis* (A) The structure of the *dcas9* and sgRNA loci. Upper: inducible *dcas9* expression system. The kanamycin-resistance gene is flanked by *lox71*/*66* sites that can be removed by Cre recombinase. Lower: double gene KD system used in this study. Two constitutive promoters of similar strength, P_*veg*_ and P_*scr*_, transcribe the first and second sgRNAs, respectively. A 26-bp barcode is inserted between the first and second sgRNAs and associated with the first sgRNA via Sanger sequencing to facilitate sequencing-based identification. Four different but equivalent transcriptional terminators ensure independent transcription of each sgRNA. The final *B. subtilis* strain contains a xylose-inducible *dcas9* gene at the *lacA* locus and the two sgRNAs at the *amyE* locus. (B) Identity of the envelope gene pairs assayed by double-CRISPRi (Table S1). (C) Schematic of the double-CRISPRi library construction method. The DNA fragments containing the sgRNAs and their associated random barcodes were cloned individually at the first position. These plasmids were then pooled, and the sgRNAs at the second position were cloned using a pooled approach (STAR Methods). (D) Correlation between RFs of two representative biological replicates. (E) Correlation between RFs of the same gene pairs with sgRNAs in the opposite order (sgRNA1-sgRNA2 versus sgRNA2-sgRNA1).

Our double-CRISPRi system is based on a xylose-inducible, chromosomally integrated, single-gene KD CRISPRi system that effects ∼100-fold KD of targeted genes.^8,26^ In double-CRISPRi, two sgRNAs targeting different genes are placed adjacent to each other on the chromosome such that double-KD strain abundances can be quantified from paired-end sequencing reads (Figure 1A). Because the loss of repeated DNA sequences is generally high (∼10^−4^/generation in *B. subtilis* and potentially higher in certain growth conditions or mutants), we minimized repeated sequences by using different but equally strong constitutive promoters, terminators, and sgRNA scaffolds for the adjacently encoded sgRNAs, thereby reducing sgRNA loss via loop-out and recombination during either the experiment or DNA sequencing.^27^ In addition to these changes at the sgRNA locus, we also replaced the erythromycin-resistance marker adjacent to *dcas9* with a kanamycin-resistance marker flanked by *lox* sequences (Figures 1A, S1A, and S1B). This change eliminates ribosome methylation by the erythromycin-resistance protein, which can affect bacterial physiology,^28^ and enables the removal and reuse of the kanamycin marker for strain construction in follow-up studies.^3,29^

Our screen targeted envelope-function genes, such as those involved in PG or TA synthesis, the later steps of membrane biogenesis, known regulators, and genes of unknown function with predicted transmembrane helices as categorized in SubtiWiki.^25^ We focused on interactions between two sets of genes (Figure 1B). The first set consists of sgRNAs targeting well-characterized envelope genes, selected essential genes, and non-targeting controls (333 total; categories 1 and 2; Figure 1B). Essential genes were targeted by mismatched sgRNAs^26^ that produce mild KD and moderate growth defects to enable the quantification of both positive and negative GIs. The second set consists of poorly characterized membrane-localized or envelope-associated genes (982 total; categories 3 and 4; Figure 1B; Table S1). Using these two sets, we aimed to identify connections between well-studied pathways and associate poorly characterized or peripheral envelope genes with established pathways.

To construct the library, we individually cloned the sgRNAs targeting the first set of genes and a random barcode into the first sgRNA position and then associated the two via Sanger sequencing (Figure 1C). Most of the sgRNAs (316/333, 95%) were successfully cloned. We next cloned sgRNAs from both sets (1,315 total) as a pool into the second sgRNA position, resulting in a library querying 415,540 (316 × 1,315) potential GIs. 93% of the potential double-CRISPRi strains were successfully constructed, and our cloning process resulted in a tight distribution of strain abundances, with ∼90% of strains within 10-fold of the median (Figure S1C). This high-quality library facilitates high-throughput screening of envelope gene GIs and provides a blueprint for double-CRISPRi library construction targeting diverse gene sets.

### Double-CRISPRi identifies high-quality GIs

To deplete targeted genes, dCas9 was induced in cells undergoing exponential growth (maintained via back dilution). Cells were sampled immediately before dCas9 induction and after 10 doublings post induction, as in our previous work.^26^ Several other time points were also collected, sequenced, and analyzed (Figure S2; Tables S2, S3, and S4). The relative fitness (RF) of each strain was calculated by comparing its relative abundance at the start and end of each experiment^26,30,31^ (Table S2; STAR Methods). Libraries were sequenced to high read depth (median read depth per strain ∼500) to enable accurate RF measurements of slow-growing strains. The RF of individual double-CRISPRi strains was highly correlated across replicates (Pearson’s *r* ∼ 0.94; Figures 1D and S3A) and with previously published single-CRISPRi experiments^26^ (Figure S3B). RFs were highly correlated between strains containing the same two sgRNAs in the opposite order (sgRNA1-sgRNA2 versus sgRNA2-sgRNA1; Pearson’s *r* ∼ 0.92; Figure 1E), indicating that the two sgRNAs were equivalently expressed despite differences in the promoters, sgRNA scaffolds, and terminators driving expression of the two sgRNAs.

**Figure 2.**
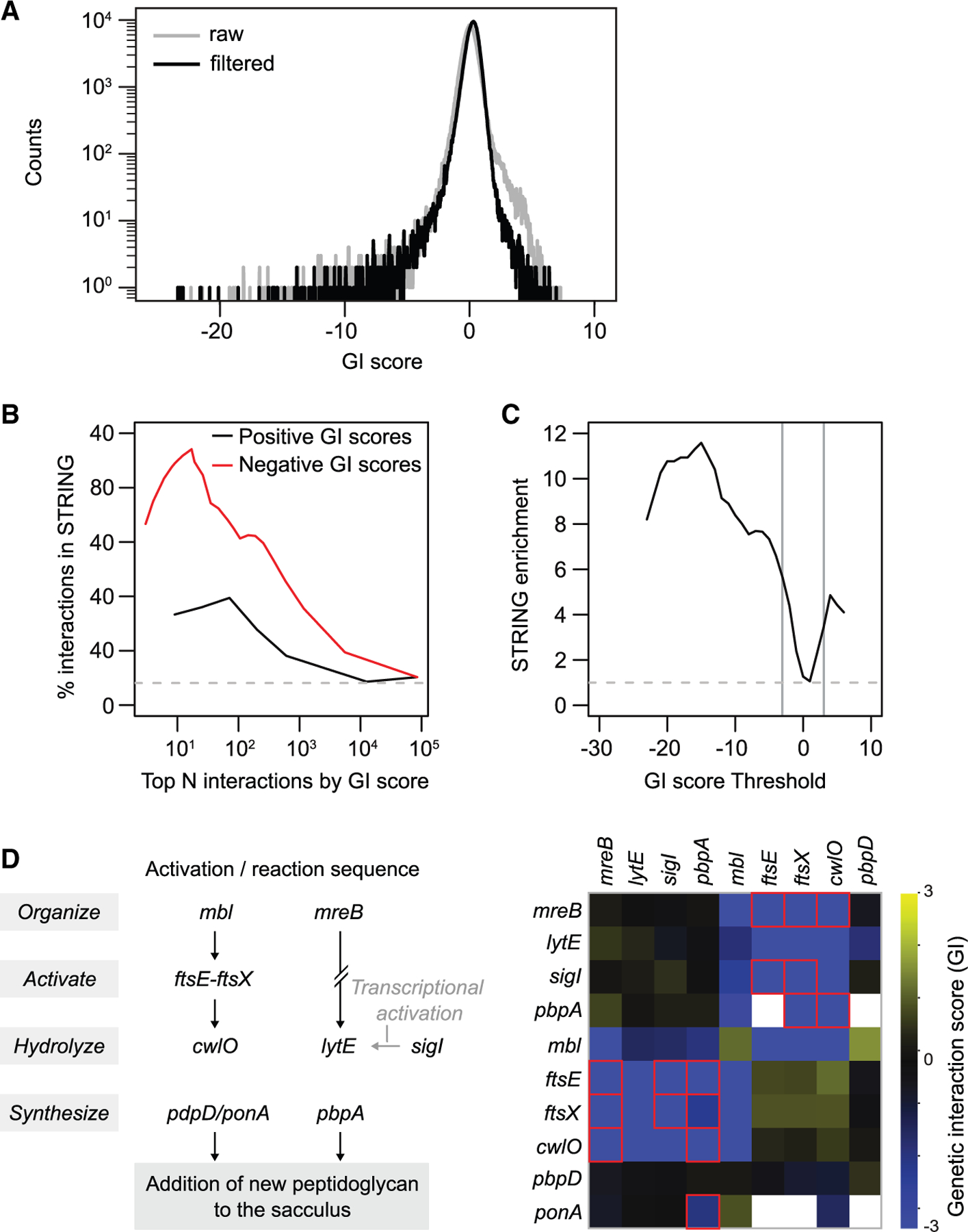
Double-CRISPRi accurately and sensitively identifies GIs Data from Table S3 (0–10 generations). (A) The distribution of GI scores in the library after 10 cell doublings with (black) and without (gray) filtering to remove sgRNAs with GIs highly correlated to interacting sgRNA sequence features (STAR Methods). Most GI scores were near zero, consistent with the hypothesis that most gene pairs do not interact. (B) Gene pairs with strongly positive (black) or negative (red) GI scores have a high proportion of gene pairs with evidence of physical or GIs from the STRING database. (C) Gene pairs with strongly positive or negative GI scores are enriched in gene pairs with evidence of physical or GIs from the STRING database. Gray lines indicate the GI score threshold at ±3. (D) Double-CRISPRi recapitulates known interactions between two cell-wall hydrolysis activation pathways. Left: schematic of the two cell-wall hydrolysis/synthesis pathways. SigI is a transcriptional activator of *lytE*, which is indicated by a gray arrow. Right: heatmap of GI scores between genes involved in these pathways. Red boxes denote previously unknown interactions identified in this screen.

To quantify GIs, we compared the RF of each double-KD strain to the RF of its two parent strains using an approach that conveys information about both the strength and statistical significance of a GI (Figure S4; STAR Methods; modified from Collins et al.^32^). Positive GI scores occur when a double-KD strain grows better than expected based on the growth defects of its parent strains (e.g., one gene is a suppressor of the other). Negative GI scores occur when a double-KD strain grows worse than expected based on the growth defects of its parent strains (e.g., the genes are synthetic sick/lethal). The final dataset (Table S3) consists of GI scores for ∼291,000 double-mutant strains that passed a set of stringent quality control standards (e.g., minimum read depth, multiple replicates, and no correlation to sgRNA sequence features; see STAR Methods). Consistent with the idea that GIs are rare,^33^ most GI scores were ∼0 (Figure 2A). The GI scores of gene pairs with a strong (absolute value of GI score |GI score| > 3) or significant (|GI score| > 2) GI in at least one replicate were highly correlated between replicates (*r* ∼ 0.79 for 2,400 interactions with |GI score| > 3; *r* ∼ 0.59 for 15,000 interactions with |GI score| > 2; Figure S5A). GI scores were also correlated between genes within the 22 operons with very strong (|GI score| > 5) GIs (median within-operon Pearson’s *r* ∼ 0.25; Figure S5B).

The large knowledge base of interactions from previous envelope-focused studies enabled us to gauge whether our quantification of GIs accurately identified known interactions. The STRING database contains known and predicted protein-protein interactions (PPIs) derived from physical, functional, and genomic associations.^34^ We found that gene pairs with high absolute GI scores (both positive and negative) were enriched in all interactions documented in STRING (Figure 2B). Indeed, those with a GI score > 3 are 3.4-fold enriched in STRING (56/202 interactions), while those with a GI score < −3 are 5.6-fold enriched in STRING (268/587 interactions) (Figure 2C). Additionally, our dataset recapitulated well-characterized synthetic-lethal phenotypes, identified GIs that are consistent with and extend known biology, and identified interactions in these pathways. For example, we identified the known synthetic lethality between the genes encoding the two cell-wall hydrolases, *cwlO* and *lytE*,^35^ and also confirmed predicted negative GIs between their activation pathways,^36^ as well as previously unreported negative GIs between hydrolases and PG synthases (e.g., *pbpA*/*cwlO* and *pbpA*/*ftsEX*) that suggest an intimate connection between PG hydrolysis and synthesis (Figure 2D). While known and expected GIs are significantly enriched in our dataset, we also identified many previously unknown high-confidence interactions that further illuminate cell envelope function. These interactions identify new partners for well-studied genes such as the essential actin homologs *mreB* and *mbl* (Figure S5C), as well as for less studied genes, indicating that our dataset can function as an engine for discovery (Table S3).

### Correlated profiles of GIs identify interacting genes

**Figure 3.**
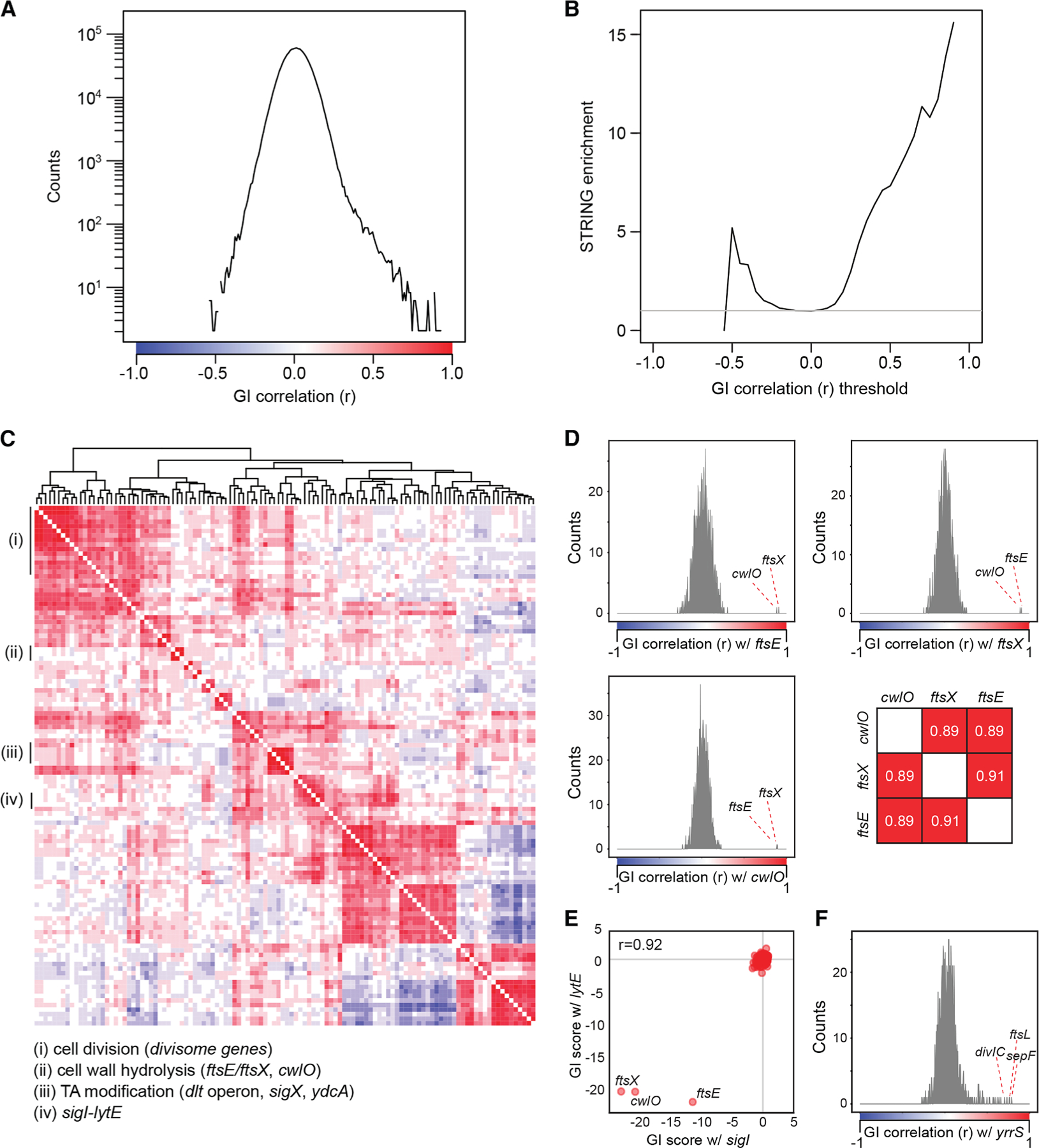
Correlated GI scores identify co-functioning gene pairs Data from Tables S3 and S4 (0–10 generations). (A) The distribution of GI score correlations between sgRNAs at the second position. (B) Correlated (and anti-correlated) gene pairs are enriched in those with evidence of physical or GIs from the STRING database. (C) Clustered heatmap of genes with at least one strong correlation (>0.5) reveals co-functioning genes. Colors indicate the Pearson correlation from (A). The uncharacterized gene *ydcA* clustered with genes involved in TA modification, likely due to a polar effect (Note S1). (D) Histograms of the correlation scores of *ftsE*, *ftsX*, and *cwlO* showing specific interactions between these three genes. (E) GI scores for all genes paired with *lytE* (*x* axis) and *sigI* (*y* axis). (F) Histogram of correlations between all genes and *yrrS* suggests a role for *yrrS* in cell division.

A gene’s pattern of GIs can be used to provide additional insight into its function by providing quantitative phenotypes that can be compared collectively to identify functionally related genes,^14,37^ analogous to the interpretation of correlated chemical sensitivities in chemical-genomics screens^4,8,38^ (Figure 3A). Consistent with this idea and with analyses in yeast^37^ and human cells,^14^ we found that gene pairs with highly correlated GI profiles were enriched in previously discovered interactions (Pearson’s *r* > 0.5, ∼7.3-fold enriched, 141/300 interactions in STRING; Figure 3B; Table S4). Hierarchical clustering of the matrix of GI score correlations distinguished cell division, cell-wall hydrolysis, and other envelope processes (Figure 3C).

Further analysis revealed three biologically relevant reasons for highly correlated gene pairs. First, genes encoding proteins in the same pathway exhibited highly correlated GI profiles. For example, FtsE and FtsX are required for the activity of the CwlO PG hydrolase.^39^ *ftsE*, *ftsX*, and *cwlO* exhibited highly correlated GI profiles with each other (*r* > 0.88), but not with other genes (the next strongest correlation was <0.31; Figure 3D). Moreover, the three genes had no strong GIs with each other (|GI score| < 1.3). Second, some sigma factors exhibited GI profiles highly correlated with those of genes in their regulon. For example, SigI has a small regulon that includes *lytE*^40^; *sigI* and *lytE* profiles were highly correlated (*r* > 0.92; Figure 3E). Finally, GI profiles were highly correlated among members of functional protein complexes such as the divisome^41^ (Figure 3C). These correlations suggested a role for the poorly characterized gene *yrrS* in cell division based on strong correlations to the GI profile of known cell division genes such as *sepF*, *ftsL*, and *divIC* (*r* > 0.7; Figure 3F). Taken together, these data indicate that correlated GI score profiles provide additional insight into the function of envelope genes.

### GI analysis reveals distinct functions of paralogous or isofunctional gene pairs

**Figure 4.**
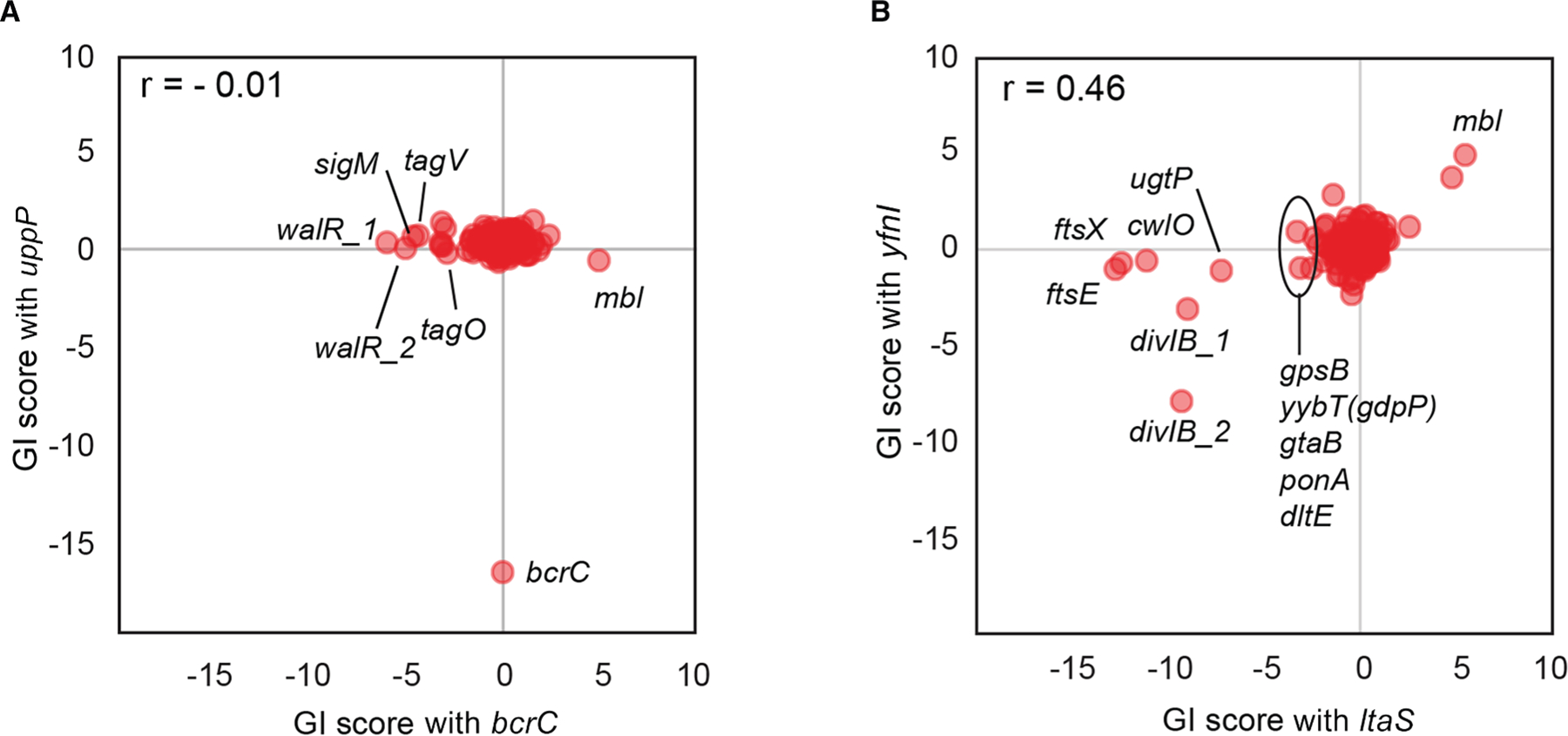
Distinct functions of paralogous/isofunctional genes Data from Table S3 (0–10 generations). (A) GI scores for strains containing *bcrC*- or *uppP*-targeting sgRNAs. *walR_1* and *walR_2* represent different sgRNAs targeting *walR*. (B) GI scores for the strains containing *ltaS* or *yfnI* targeting sgRNAs. *divIB_1* and *divIB_2* represent different sgRNAs targeting *divIB*.

Duplication and divergence of genes are major drivers of evolution, and as a result, paralogous genes are common in bacteria,^42^ as are isofunctional (but non-homologous) genes. However, our understanding of the shared and distinct functions of such pairs is often incomplete. We examined the GI profiles of 3 paralogous or isofunctional gene pairs to assess their degree of functional divergence: the non-homologous undecaprenyl pyrophosphate phosphatases *bcrC* and *uppP* (Figure 4A), the paralogous lipoteichoic acid (LTA) synthases *ltaS* and *yfnI* (Figure 4B), and the actin homologs *mreB* and *mbl* (Figure S5C).

*bcrC* and *uppP* each encode an undecaprenyl pyrophosphate phosphatase, a key enzyme in the lipid II cycle, and have approximately equivalent transcript levels.^43^ BcrC has been proposed to be the major enzyme in *B. subtilis*.^44^ Consistent with this designation, Δ*bcrC* but not Δ*uppP* mutants exhibited a slow-growth phenotype.^45^ *uppP* and *brcC* exhibited sharply divergent GI profiles. *uppP* exhibited only one strong GI (synthetic lethal with *bcrC*^46^), whereas *bcrC* exhibited many strong GIs, including strong negative interactions with *sigM*, *tagO*, and *tagV* (Figure 4A). Since *sigM* becomes essential under undecaprenyl phosphate (Und-P)-limiting conditions,^47^ the strong negative GI between *bcrC* and *sigM* suggests that BcrC depletion significantly reduces Und-P levels. The strong negative GIs with the most upstream gene involved in wall TA (WTA) synthesis, *tagO*, and the phosphotransferase gene for WTA attachment, *tagV*, could result from disruption of the lipid II cycle, as these two enzymes use or produce Und-P after their catalytic reactions.^48,49^ Interestingly, in *E. coli*, the roles of the two Und-Pases are reversed: *uppP* is responsible for 75% of undecaprenyl pyrophosphate phosphatase activity, while *bcrC* is considered a minor enzyme.^50^

The *ltaS* and *yfnI* paralogs are LTA synthases, with stress-activated *yfnI* producing longer LTAs than *ltaS*. While there is overlap in their GI profiles (e.g., strong negative GIs with *divIB*, an essential divisome member; Figure 4B), there are also differences. *ltaS* (but not *yfnI*) KD exhibited strong negative GIs with *ftsEX* and *cwlO*, suggesting that the LTA polymers produced by each paralog have differential effects on the PG elongation machinery (Figure 4B). *ltaS* (but not *yfnI*) KD also showed weak but consistent negative GIs with the *dlt* genes (Figure 4B). We validated these phenotypes using Δ*ltaS*/Δ*dlt* and Δ*yfnI*/Δ*dlt* double-deletion mutants (Figure S6). The growth defect of the Δ*ltaS*/Δ*dlt* strain, in which almost all LTAs are synthesized by YfnI, suggests that LTAs produced by YfnI require D-alanylation mediated by the *dlt* genes^51^ for full functionality, whereas those produced by LtaS do not.

**Figure 5.**
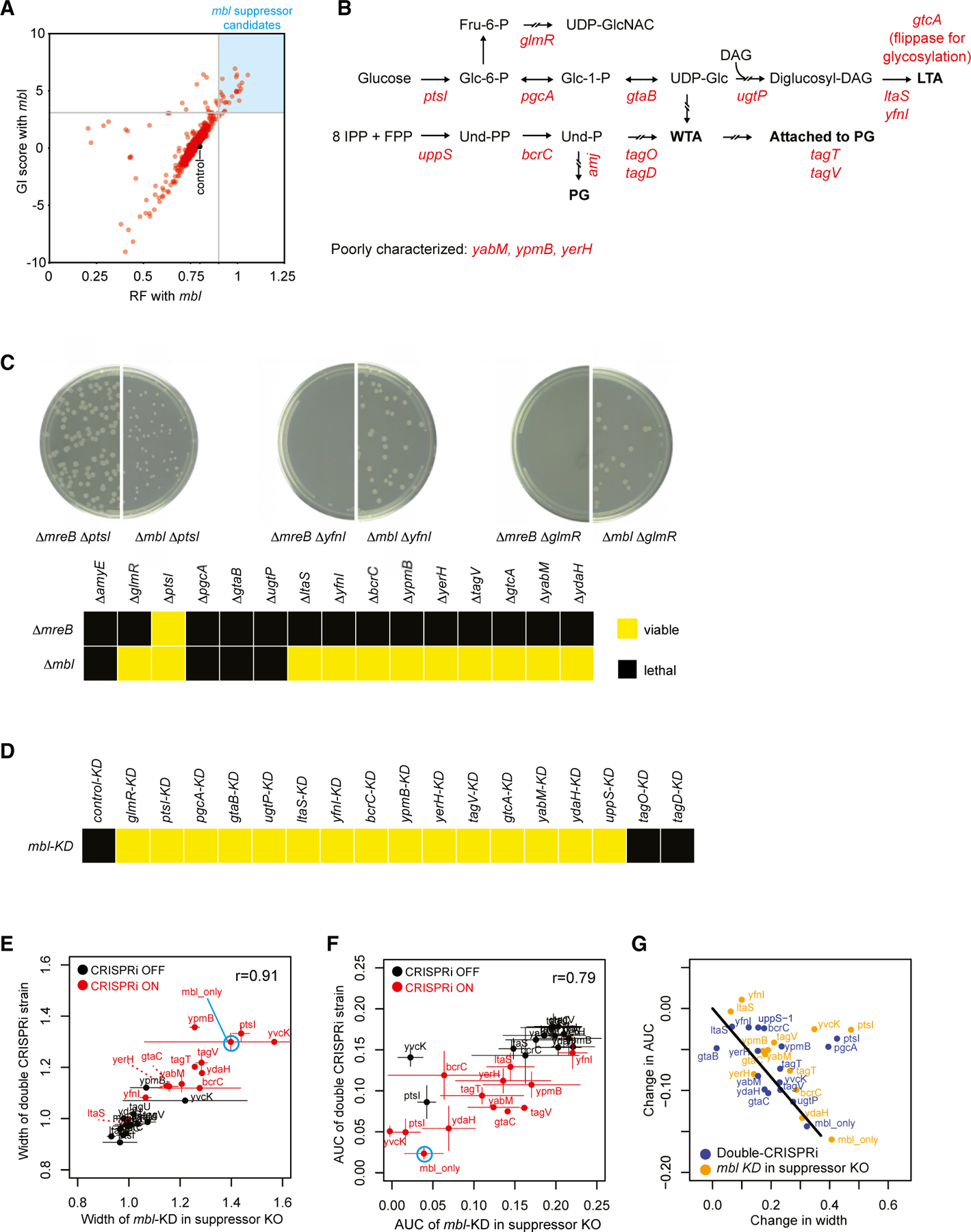
GIs disentangle the roles of *mbl* and *mreB* in cell envelope homeostasis Data from Tables S2 and S3 (0–10 generations). (A) *mbl* suppressors for follow-up are indicated in the blue quadrant. Suppressors chosen have an RF > 0.9 and a GI score > 3 relative to the mean RF ∼ 0.78 (gray dot) of *mbl*-control strains in *mbl* double-CRISPRi strains. (B) *mbl* suppressors were involved in various aspects of WTA, LTA, and PG synthesis. *glmR* is a positive regulator of UDP-N-acetylglucosamine (UDP-GlcNAC) synthesis. UDP-GlcNAC is a precursor for PG and other metabolites. (C) Viability of Δ*mbl* or Δ*mreB* in *mbl*-specific and common suppressor backgrounds. *mbl::kan* or *mreB::kan* fragments were transformed into each strain and visualized after 16 h (STAR Methods). Top: representative images of the transformation plates of Δ*ptsI*, Δ*yfnI*, and Δ*glmR* strains. Bottom: summary of viability of all double mutants, with viable pairs indicated by yellow squares. (D) Viability of individually constructed double-CRISPRi KD strains of all *mbl*/suppressor pairs. Viable KD pairs are indicated as in (C). (E) Comparison of cell width changes obtained from microscopy in two groups of strains: set 1, the double-KD set (CRISPRi KD of each *mbl* suppressor + KD of *mbl*); set 2: the KD/deletion set (*mbl* KD + suppressor deletion). In CRISPRi OFF strains (black dots), Mbl is present, and suppressors are either present (set 1) or deleted (set 2). In CRISPRi ON strains (red dots), Mbl levels are highly reduced, and suppressor levels are either highly reduced (set 1) or absent (set 2). Note that the *mbl*-only strain has a single sgRNA targeting *mbl*. Although all measurements have error bars, in general, the error bars for set 1 (vertical) are much smaller than those for set 2 (horizontal). (F) Comparison of cell growth measurements obtained from bulk cultures, calculated as AUC using strain sets and CRISPRi induction as in (E). (G) Quantitative comparison of the extent of rescue of cell morphology and cell growth using strains and data in (E) and (F). Abbreviations: Fru-6-P, fructose-6-phosphate; UDP-GlcNAC, UDP-N-acetylglucosamine; Glc-6-P, glucose-6-phosphate; Glc-1-P, glucose-1-phosphate; UDP-Glc, UDP-glucose; DAG, diacylglycerol; IPP, isopentenyl pyrophosphate; FPP, farnesyl pyrophosphate; Und-PP, undecaprenyl pyrophosphate; Und-P, undecaprenyl phosphate; PG, peptidoglycan.

MreB and Mbl are well-studied essential paralogs that function in cell shape determination through regulation of cell-wall elongation.^52^ Whereas *mreB* is almost universally conserved in rod-shaped bacteria, additional *mreB* homologs such as *mbl* are found exclusively in gram-positive bacteria.^53^ *mbl* or *mreB* can be deleted in the presence of excess Mg^2+^, which stabilizes the cell envelope and inhibits the activity of LytE and perhaps other PG hydrolases, but knockout strains exhibit morphological defects.^54–56^ As expected, *mreB* and *mbl* exhibited a strong negative GI in our screen (Figures 2D and S5C). Additionally, *mreB* and *mbl* both had negative GIs with *ftsE*, *ftsX*, and *cwlO*, confirming their synergistic role in guiding the elongation machinery and controlling the activity of cell-wall hydrolases (Figures 2D and S5C). Although we identified many positive (suppressive) GIs for *mbl*, including known suppressors such as *ltaS*,^57^ we found no GIs for *mreB* (Figures 5A, blue quadrant, and S5C). First, disrupting genes involved in LTA synthesis, including the major and minor LTA synthases (*ltaS* and *yfnI*), the LTA glycosylation protein (*gtcA*), and genes involved in TA precursor synthesis (*pgcA*, *gtaB*, and *ugtP*), rescued *mbl* KD (Figure 5B, top pathway). Second, many genes involved in WTA synthesis (*tagO* and *tagD*), lipid carrier cycling (*uppS*, *bcrC*, and *amj*), and attachment (*tagT* and *tagV*) had positive GIs with *mbl*, suggesting a previously unrecognized role for WTAs in regulating cell elongation (Figure 5B, bottom pathway). Finally, genes involved in sugar metabolism (*ptsI* and *glmR*) and several poorly characterized genes (*ypmB*, *yerH*, and *yabM*) had positive GIs with *mbl*. Taken together, these data suggest that GI analysis can disentangle the shared and unique functions of partially redundant genes.

### Dissecting the role of *mbl* in TA synthesis

Our observation that *mbl*, but not *mreB*, has positive GIs with genes in LTA/WTA synthesis and other processes could result from two technical considerations. First, *mreB* was targeted by a mismatched sgRNA (partial KD, mild phenotype), making it harder to detect positive interactions. Second, KD of *mreB* likely affects the expression of downstream essential genes in its operon (*mreC*, *mreD*, and other cell division genes), which may influence its GIs. We therefore validated that the suppressors we identified were *mbl*-specific using an orthogonal approach. We tested whether Δ*mbl* or Δ*mreB* alleles could be transformed into strains carrying a deletion of each putative *mbl* suppressor gene described above, a strategy that completely eliminates *mreB* expression while allowing expression of downstream genes. In our growth/media conditions, Δ*mbl* could be transformed into almost all suppressor gene deletion strains, but Δ*mreB* could be transformed only into Δ*ptsI*, a known *mreB* suppressor^58^ (Figure 5C), validating these suppressors as *mbl-*specific and implying some distinct roles for *mreB* and *mbl*. Additional evidence for distinct roles comes from their differential interaction with *glmR*: *mbl* essentiality is suppressed by deleting *glmR* (Figure 5C), whereas *mreB* essentiality is suppressed by overexpressing *glmR*.^59^ Although double-deletion strains of *mbl* and suppressor genes were viable in the exponential phase, survival into the stationary phase required activation of PG synthesis systems (Note S2). Taken together, these data validate the results of our double-CRISPRi screen and greatly expand the known set of *mbl*-interacting processes, adding both WTA synthesis and genes of unknown function.

The previously characterized *mbl* suppressor, *ltaS*, restores wild-type growth and reverses the cell widening observed in Δ*mbl* strains prior to lysis.^57^ We tested whether the same was true of our newly identified *mbl* suppressors, using two additional strain sets. The first set consisted of individually reconstructed double-KD pairs of *mbl* and the individual suppressors. We used this set because three suppressors (*pgcA*, *gtaB*, and *ugtP*) could not be reconstructed via knockout (Figure 5C). Our second set contained suppressor deletions coupled with *mbl* KD to account for potential polar effects. For each strain, we obtained cell width measurements using microscopy (Figure 5E) and growth measurements from bulk cultures, calculated as area under the growth curve (AUC, Figure 5F). Both mean cell width (*r* > 0.90, *p* < 10^−10^) and growth AUC (*r* > 0.78, *p* < 10^−6^) were similar between the two sets. The degree of growth and morphological rescue were correlated for most strains (Figure 5G). However, a few genes (*pgcA*, *ptsI*, and *glmR*) rescued only growth, consistent with a recent study showing that suppressing the lethality of *mbl* deletion does not require morphological compensation.^60^ Taken together, these data suggest a role for *mbl* in WTA and LTA synthesis and attachment that impacts growth and cell-shape determination and is not shared with *mreB*.

### Identification of additional genes involved in cell division

**Figure 6.**
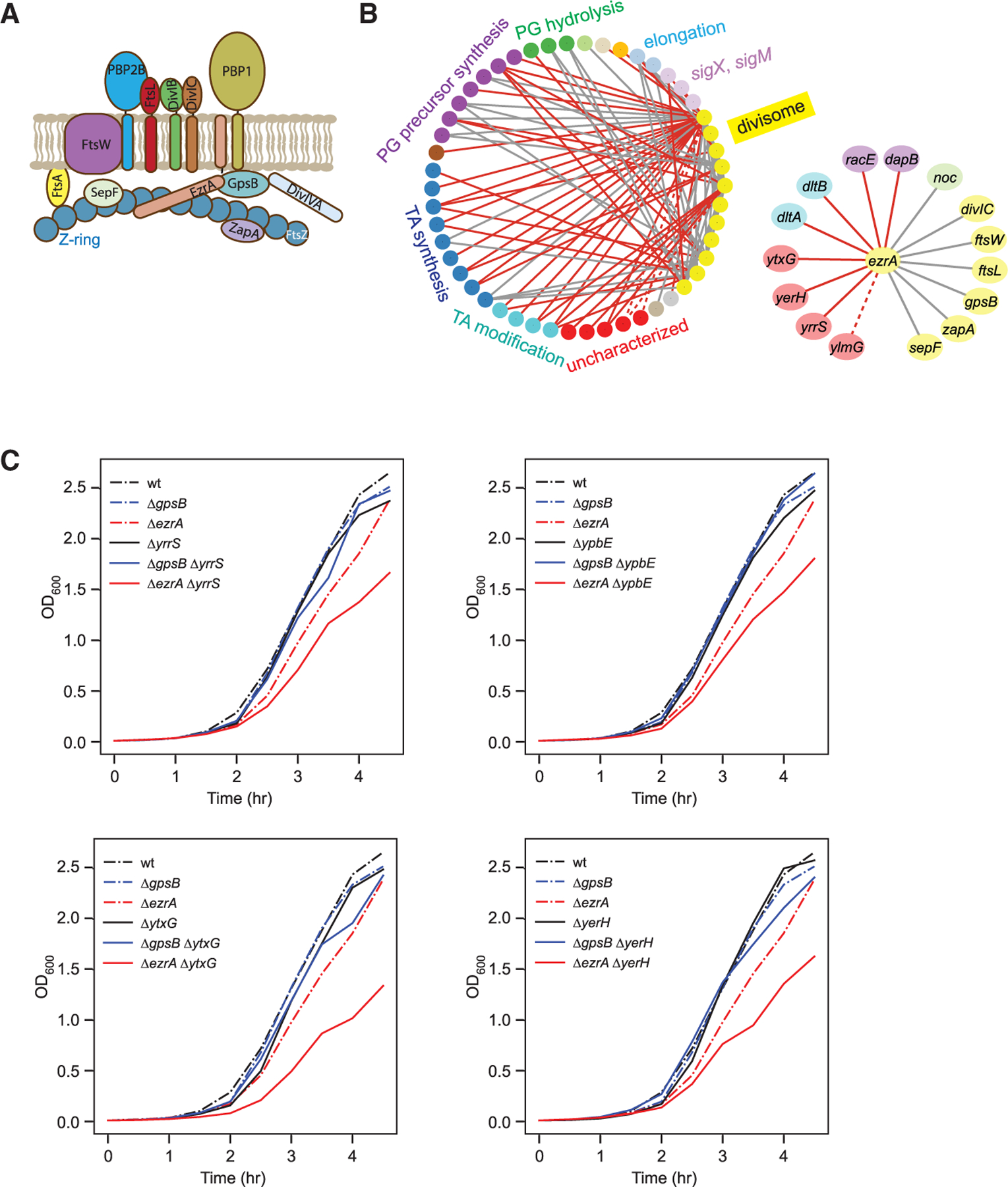
Double-CRISPRi identifies additional players in *B. subtilis* cell division (A) Schematic of *B. subtilis* divisome complex showing the FtsZ ring and associated cell-wall synthesis machinery.^41^ (B) Left: schematic of intra- and inter-GI network of divisome genes. Right: GI network of *ezrA*. Gray lines indicate known interactions, and red lines indicate previously unknown interactions identified in this screen. The dotted line between *ezrA* and *ylmG* indicates a false negative GI resulting from KD of *sepF* in the same operon due to the polar effect of CRISPRi. (C) Deletion of potential cell division genes (*yrrS*, *ypbE*, *ytxG*, and *yerH*) resulted in a synthetic growth phenotype with Δ*ezrA*, but not with Δ*gpsB*. Two independent experiments were performed, and representative data are shown here.

Bacterial cell division is a highly orchestrated process in which constriction driven by the divisome machinery must be coordinated with cell-wall synthesis to avoid lysis.^61–63^ To divide, cells form an FtsZ ring (Z-ring) at the site of the future septum that is used as a platform to assemble the divisome (Figure 6A), and the membrane constricts as PG is synthesized to form the septum and separate the daughter cells.^41,64,65^ Since many cell division genes are essential, their GIs cannot be explored using a method dependent on knockouts. Our double-CRISPRi screen targeted essential genes with mismatched sgRNAs that reduce but do not eliminate gene expression, enabling us to identify both positive and negative GIs of essential genes. In our dataset, many divisome genes formed a highly connected network composed of strong negative GIs (Figures 6B and S7), consistent with the known co-dependence of these genes in cell division.^41,64,65^ Moreover, we identified strong previously un-identified negative GIs between divisome genes and genes involved in PG precursor synthesis, PG remodeling, and TA synthesis/modification, as well as ECF sigma factors (SigX and SigM), reflecting the characterized activities of divisome proteins.^64,65^ By searching for additional genes that exhibited strong GIs or correlated GI profiles with known division genes, we also identified several additional players in cell division, including the uncharacterized genes *yrrS*, *ytxG*, and *yerH*, whose potential negative interaction with *ezrA* was revealed by our dataset (Figure 6B).

EzrA is a negative regulator of Z-ring formation. In its absence, *B. subtilis* cells can have multiple Z-rings concurrently at the cell poles and mid-cell.^65,66^ EzrA also works with GpsB to recruit PBP1 to the division septum^67^ and activate PrkC.^68^ Our dataset verified the previous finding that *ezrA* and *gpsB* are synthetic sick,^67^ and we additionally found strong negative GIs between *ezrA* and its known interaction partners *sepF*^69^ and *zapA*.^70^ Despite strong negative GIs with *ezrA*, neither *yrrS*, *ytxG*, nor *yerH* exhibited strong negative GIs with these known EzrA partners, raising the possibility that these uncharacterized genes function with one of the EzrA partners. Consistent with this hypothesis, YrrS in *B. subtilis* and YtxG in *S. aureus* have been reported to physically interact with GpsB.^71,72^

To validate the GIs of these uncharacterized genes, we constructed and analyzed deletion strains of *yrrS*, *ytxG*, and *yerH* as well as *ypbE* (which was missing from our screen due to low sequencing read depth but has a similar PPI profile to that of *yrrS*^71^ in a Δ*ezrA* strain). Using these double mutants, we found that, as measured by growth, all four y-genes exhibited negative GIs with *ezrA* but not *gpsB*, consistent with the results of our pooled screen (Figure 6C). Since YrrS and YpbE are known to bind to each other,^71^ we also asked whether they interact synergistically with *ezrA*. Indeed, although the *yrrS*/*ypbE* double mutant exhibited no significant growth phenotypes, the *yrrS*/*ypbE*/*ezrA* triple deletion mutant was much sicker than predicted (Figures S8A and S8B). The interaction was specific to *ezrA*: *yrrS*/*ypbE* double mutants did not exhibit negative GIs with other *ezrA*-interacting cell division genes such as *gpsB*, *sepF*, and *zapA* (Figure S8B). These proteins may also have additional roles in division, as each had distinct but uncorrelated GIs (Table S4; Note S3).

### Single-cell imaging reveals cell division phenotypes for *yrrS*, *ypbE*, *ytxG*, and *yerH*

Each of the y-genes exhibited negative interactions with *ezrA*, and YrrS, YpbE, and likely YtxG bind GpsB, suggesting that GpsB is scaffolding their activities. Therefore, we examined how the phenotypes of cells with *ezrA* knocked down in combination with deletion of *yrrS*, *ypbE, ytxG*, or *yerH* compared with known *ezrA* mutant phenotypes and *ezrA*-KD/Δ*gpsB* phenotypes. We acquired phase-contrast images, computationally segmented the contours of thousands of cells, and quantified contour dimensions to detect filamentation. FM4–64 labeling of the same cells was used to visualize the membrane cross-bands that demarcate individual cell boundaries within an apparent filament, and DAPI labeling was used to capture nucleoid morphology and localization. In separate cells, we also monitored Z-ring localization and kinetics using a NeonGreen-FtsZ fusion^73,74^ (STAR Methods).

**Figure 7.**
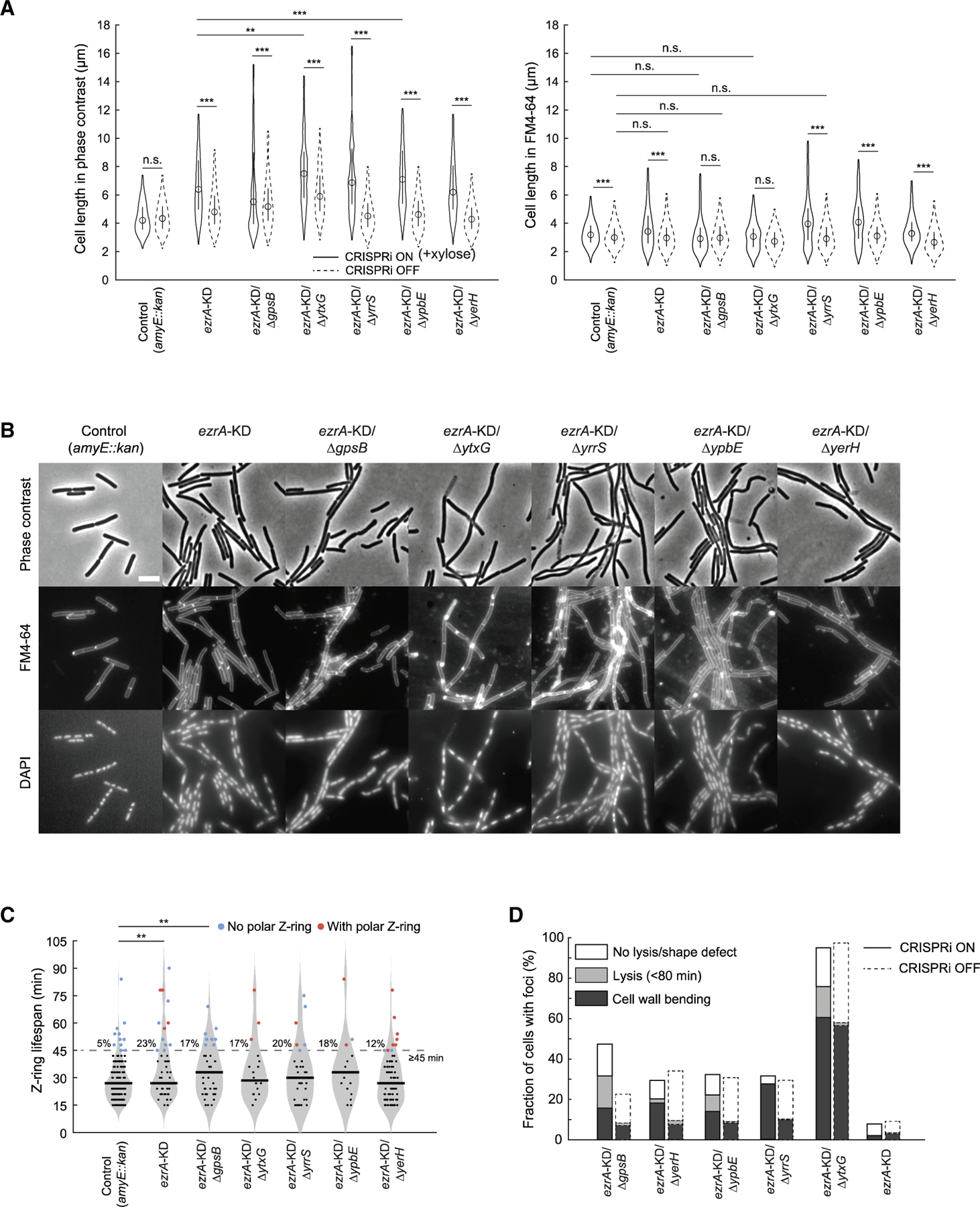
Phenotypes of knockout mutants of several uncharacterized genes in combination with *ezrA* depletion (A) Depletion of EzrA causes apparent filamentation in Δ*gpsB* and several previously uncharacterized mutants (Δ*yerH*, Δ*ypbE*, Δ*yrrS*, and Δ*ytxG*) compared with *ezrA* KD alone or gene knockouts without *ezrA* KD, as indicated by phase-contrast microscopy (left). However, the length of cell compartments delineated by FM4-64-labeled membrane staining showed limited increase compared with the control strain, suggesting unperturbed Z-ring localization (right). All images were acquired in a single imaging session involving one biological replicate. *n* > 200 cells were quantified for each distribution. Statistical significance was calculated using Welch’s t test. **p < 0.01, ***p < 0.001. (B) The elongated mutant cells caused by *ezrA* KD are separated into shorter compartments based on FM4-64-labeled division planes. In all mutants, cell division planes occurred away from the DAPI-labeled nucleoid, as normally seen in wild type. Scale bar: 5 μm. (C) *ezrA* KD alone or together with Δ*gpsB*, Δ*yrrS*, Δ*ypbE*, Δ*ytxG*, or Δ*yerH* increased the fraction of Z-rings with prolonged lifespan, leading to persistent Z-rings at the adjacent new poles after cell division in all mutants except *ezrA-*KD/Δ*gpsB*. *n* ≥ 17 Z-rings were tracked for each strain. Statistical significance was calculated using Welch’s t test. (D) The fraction of cells containing bright foci and those developing shape defects or undergoing lysis is dependent on the deletion of specific genes and KD of *ezrA*, underscoring the impact of GIs on cell division defects. The fraction of cells with bright foci increased in double mutants regardless of induction of *ezrA* KD, likely because basal KD of *ezrA* combined with each gene deletion is sufficient to increase this phenotype. *n* > 50 cells were counted for each combination of strain and condition, except for *ezrA* Δ*gpsB* with induction of the CRISPRi system (*n* = 19) due to relatively low cell viability.

EzrA binds to and regulates FtsZ, thereby influencing Z-ring formation and construction of the divisome. Previous studies showed that Δ*ezrA* cells form apparent filaments but largely maintain normal nucleoid segregation and are able to septate.^66^ We found that cells with *ezrA* knocked down alone or in combination with Δ*yrrS*, Δ*ypbE*, Δ*ytxG*, or Δ*yerH* mirrored these phenotypes, largely exhibiting proper nucleoid placement and septation, although like *ezrA*-KD/Δ*gpsB*, the double mutants exhibited longer apparent filaments than *ezrA*-KD alone (Figures 7A and 7B; *p* < 0.001 for all strains, Welch’s t test). Likewise, *ezrA* knocked down alone or in combination with Δ*yrrS*, Δ*ypbE*, Δ*ytxG*, or Δ*yerH* exhibited prolonged Z-ring lifespan (12%–23% of Z-rings persisted for ≥45 min; Figure 7C) due to delayed disassembly, resulting in persistent Z-rings at the adjacent new poles after cell division completed (Figure S9). The observation that Z-ring formation along the cylindrical portion of the apparent filaments occurred at intervals demarcating similar length compartmentalization as the FM4–64 signal reinforced our conclusion that the cell division machinery is properly localized in all double mutants. Moreover, when an intermediate compartment lysed (visualized as loss of phase contrast), adjacent compartments retained phase contrast (Figure S10), indicating functional compartmentalization. Taken together, these results indicate that like *ezrA*-KD alone, nucleoid segregation and cell division in the double mutants were not grossly disrupted, resulting in cellular compartments of length comparable to cells without *ezrA* KD.

Although GpsB is present at all sites of PG synthesis, the Δ*gpsB* phenotypes that have been identified in Δ*ezrA*/Δ*gpsB* cells are septum related, likely reflecting the fact that loss of both adaptor proteins results in substantial dysregulation of the highly coordinated processes necessary for normal cell-wall synthesis during cell division.^67,75^ Thus, we expected that *ezrA* KD in combination with Δ*yrrS*, Δ*ypbE*, Δ*ytxG*, or Δ*yerH* would exhibit a subset of the phenotypes of *ezrA*-KD/Δ*gpsB* cells, although the severity and types of defects would likely be mutant specific due to removing specific functions rather than the protein that coordinates and directs their activities. We used this framework to interpret the shared phenotypes of all *ezrA*-KD double mutants. The functional cellular compartmentalization in all mutants suggested that the apparent filamentation was due to post-septation processes such as polar maturation. During polar maturation, nascent PG with a pentapeptide stem is converted to mature PG with a tetrapeptide concomitant with cell separation, and this process is slowed in *ezrA*-KD/Δ*gpsB* cells.^75^ Slower polar maturation would increase the length of the apparent filaments by increasing the residency time of each cell in the filament.

We also noted several morphological defects in Δ*gpsB*, Δ*yrrS*, Δ*ypbE*, and Δ*yerH* cells upon *ezrA* KD. FM4–64 staining revealed that a subset of cells contained membrane foci, and the fraction of cells with severe morphological changes increased upon *ezrA* KD (Figure 7D). Moreover, upon *ezrA* KD, time-lapse imaging revealed that these foci were frequently localized near areas that developed morphological defects such as bending or lysis (Figure S11A). Additionally, apparently fully formed membrane cross-bands and invaginations sometimes disappeared (Figure S11B) and were replaced by uniform swelling nearby (*ezrA*-KD/Δ*gpsB*) or by a sharp bend (*ezrA*-KD/Δ*yrrS* and *ezrA-*KD/Δ*yerH*) (Figure S11B). Finally, as previously reported,^72^ virtually all Δ*yxtG* cells exhibited foci regardless of *ezrA* KD (Figure 7D), with an increased fraction lysing upon KD. The membrane invaginations causing these foci have been attributed to incorrect recruitment of septal factors,^72,76^ suggesting that pre-existing defects in PBP localization are exacerbated upon *ezrA* KD. Defects in septal PG synthesis were among the first phenotypes documented for Δ*ezrA*/Δ*gpsB*^67^ and are likely a consequence of loss of GpsB-dependent localization of PG-related enzymes and other factors. The mutant phenotypes we observed likely reflect loss of at least one such enzymatic activity or imbalanced synthesis from the remaining GpsB-associated proteins. Bulging has also been ascribed to the failure of a Δ*ezrA*/Δ*gpsB* strain to complete cell pole maturation.^67^

Taken together, our data demonstrate the ability of GI analyses to reveal additional genes involved even in well-studied processes like cell division and highlight the diversity of phenotypes that can emerge from disruption of the division machinery. Additionally, our single-cell analyses highlight the relationship of our mutant phenotypes to those of *ezrA-*KD/Δ*gpsB*, indicating that these mutants participate in GpsB-mediated processes impacting septum maturation.

### Perspective

Here, we introduced double-CRISPRi, an experimental and analytical approach for high-throughput CRISPRi-based GI mapping in bacteria. We used double-CRISPRi to perform genome-scale GI mapping of envelope-function genes (including essential genes) in the model bacterium *B. subtilis*. Our focus on mapping interactions between cell envelope-related genes allowed us to validate many of our findings using the vast existing knowledge base. This GI map serves as a broad resource for further characterization of envelope gene function, and our experimental and analytical framework will enable future GI mapping efforts in *B. subtilis* and other diverse bacteria.

Our analysis of GIs in the *B. subtilis* cell envelope supports three major conclusions. First, we established double-CRISPRi as a powerful tool for understanding bacterial gene functions and pathway connections. The GIs accurately identified both known and novel functional partners of many genes, enabling us to connect diverse processes and dissect complex pathways. This success was exemplified by our studies of *mbl* and *mreB*, which guide the elongation machinery. This machinery contains a pair of synthetic-lethal PG hydrolases, *lytE* and *cwlO*, which maintain the balance between PG synthesis and disassembly that is essential for cell proliferation.^77^ Although previous studies found that MreB and Mbl differentially activate these hydrolases,^36^ our study additionally uncovered an extensive network of GIs involving these genes. Moreover, our study identified extensive GIs between *mbl* (but not *mreB*) and many other processes, including LTA and WTA synthesis, the regulation of metabolism, and cell division, that will motivate future studies. Our finding that *mbl* and other elongasome components genetically interact with division genes such as *divIVA*, *divIB*, *sepF*, *ftsL*, and *ftsA* is supported by a concurrent double-CRISPRi screen in *Streptococcus pneumoniae*, which found and validated negative GIs between *divIB*/*divIC* and many components of the elongasome.^78^

Second, we established the ability to identify additional members of essential cellular machines. Our screen leveraged mismatch-CRISPRi^26^ to design sgRNAs that target essential genes with intermediate efficacy, resulting in single mutants with quantifiable growth rates that enabled the identification of both positive and negative GIs. A notable example of this ability was the identification of additional players in the well-characterized and intensively studied process of cell division. Cell division genes, including many essential genes such as *divIB* and *ftsL*, formed a highly interconnected network of GIs. We identified and characterized four genes connected to this cluster, highlighting the utility of GI mapping for discovering the complete network of divisome interactions. Single-cell imaging of these mutant strains revealed division defects unrelated to the inhibition of septum formation, with morphological defects suggesting overstabilization of the division machinery and mislocalization of growth at sites of intended septa, as has been observed in Δ*ezrA*/Δ*gpsB* double mutants.^67^ Our GI data support a role for these genes in cell division via localization of PBP1 (Note S3). Three of these genes (*yrrS*, *ypbE*, and *yerH*) are conserved primarily in *Bacillus* species and closely related genera, suggesting a specialized function in the division machinery of these species. However, *ytxG* is broadly conserved in both rod-shaped and coccoid Firmicutes and exhibits distinct phenotypes in each. Together, these data suggest that while the core cell division machinery is highly conserved,^65^ accessory factors and PPIs can differ across taxa. Future double-CRISPRi studies in diverse bacteria will reveal how the cell division machinery has been adapted to different cell shapes (rod, cocci, and spiral), modes of cell-wall growth (symmetric division and apical growth), and bacterial lifestyles.

Third, at a broader level, our screen begins to reveal the nature and frequency of GIs in bacteria, which informs and constrains future studies. As expected based on GI studies in yeast,^13^ essential and well-characterized genes (gene set 1) exhibited more GIs (∼3.8 on average with |GI score| > 2) than uncharacterized genes (gene set 2: ∼0.5 on average with |GI score| > 2), suggesting that the former set may function as network hubs. This result highlights the utility of targeting essential genes with mismatched sgRNAs and of ensuring high library coverage and sequencing depth to accurately quantify strong growth defects. Moreover, the surprising number of inter-process connections argues for selecting broad gene sets rather than focusing on single processes in future studies.

Our study provides a valuable dataset for deciphering cell envelope gene function in *B. subtilis* as well as a blueprint for studies in other bacteria. The double-CRISPRi approach is a robust tool for deeper and broader study of bacterial GI networks, which can illuminate new biology and enable rational design of antibiotic combination therapies. Double-CRISPRi libraries can be (1) used to conduct chemical-genomic screens that reveal multi-partite interactions and phenotypes for highly redundant genes, (2) combined with mobile-CRISPRi^79^ to study GIs in diverse bacteria, and (3) (with modifications, see limitations of the study) be scaled to target all genes in a bacterial genome. Additionally, our current envelope-focused double-CRISPRi library can be combined with high-throughput microscopy or flow-cytometry approaches^72,80,81^ to assay cell shape, size, and other non-growth-related phenotypes. Double-CRISPRi will serve as an important tool for closing the gene sequence-function gap across bacterial species.

### Limitations of the study

The substantial insights into cellular connectivity enabled by our double-CRISPRi method motivate future efforts to target the entire genome. In our study, we individually cloned the first of the two sgRNAs to ensure even representation in the double-mutant pool. However, genome-wide targeting requires pooled cloning of sgRNAs at both positions, which can be accomplished using optimized plasmids (pDCi00; Table S5). Targeting every pairwise combination of the ∼4,000 genes (∼16 million strains) in a typical bacterial genome would require growth of large-volume cultures to avoid bottlenecking and would entail proportionately higher sequencing costs. To mitigate these issues, a double-CRISPRi library could be designed to target only the first gene in an operon, relying on CRISPRi polarity to repress downstream genes.^8^ As ∼50% of bacterial genes are in operons,^82^ such a strategy would reduce library size ∼4-fold. However, only computational predictions of operon structure are available for many species. These predictions incorrectly annotate some operon boundaries and can miss (conditional) internal promoters. Indeed, the discordant GIs of operon members *pbpI* and *yrrS* are likely due to a promoter upstream of *yrrS* that is un-affected by *pbpI* KD. Therefore, it is likely prudent to target each gene individually, which should be increasingly tractable as advances in sequencing and synthesis technology reduce the associated costs.

## RESOURCE AVAILABILITY

### Lead contact

Further information and requests for resources and reagents should be directed to and will be fulfilled by the lead contact, Carol Gross (cgrossucsf@gmail.com).

### Materials availability

Strains and plasmids generated in this study are available upon request with a completed materials transfer agreement.

### Data and code availability

- Raw sequencing data (FASTQ) associated with this project have been deposited to NCBI BioProject: PRJNA1143566 and are publicly available as of the date of publication. All analyzed GI scores and correlation data are available in the paper’s supplemental table as indicated. All other data reported in this paper will be shared by the lead contact upon request.
- All original code has been deposited at https://github.com/traeki and https://github.com/horiatodor and is publicly available as of the date of publication. DOIs are listed in the key resources table.

## STAR★METHODS

### EXPERIMENTAL MODEL AND STUDY PARTICIPANT DETAILS

#### Strains and growth conditions

All strains used in this study are listed in Table S5. All *B. subtilis* strains are derivatives of the 168 strain (Bacillus Genetic Stock Center; accession number: 1A1). Cells were routinely grown in lysogeny broth (LB) medium (1% tryptone, 0.5% yeast extract, and 0.5% NaCl) at 37 °C with aeration or on LB agar plates supplemented with appropriate antibiotics at the specified concentrations (by activity) if needed: for *B. subtilis*, erythromycin (1 μg/mL), lincomycin (12.5 μg/mL), spectinomycin (100 μg/mL), chloramphenicol (6 μg/mL), kanamycin (7.5 μg/mL); for *E. coli*, carbenicillin (100 μg/mL).

### METHOD DETAILS

#### Genetic manipulation

Transformation of *E. coli* with plasmids was performed using the heat shock method or electroporation as described in the New England Biolabs (NEB) protocol (https://www.neb.com/en-us/protocols/0001/01/01/high-efficiency-transformation-protocol-c3019, https://www.neb.com/en-us/protocols/0001/01/01/electroporation-protocol-c3020).

Transformation of *B. subtilis* was performed using natural competence. Competent cells were prepared using the following protocol^3^: *B. subtilis* cells were inoculated into 3 mL of MC medium (10.7 g/L K_2_HPO_4_, 5.2 g/L KH_2_PO_4_, 20 g/L glucose, 0.88 g/L trisodium citrate dihydrate, 0.022 g/L ferric ammonium citrate, 1 g/L casamino acids, 2.2 g/L potassium glutamate monohydrate, 20 mM MgSO_4_, 300 nM MnCl_2_, 20 mg/L L-tryptophan) and incubated at 37 °C overnight with aeration. The overnight culture was diluted to an OD_600_ of 0.1 in 20 mL of competence medium (10.7 g/L K_2_HPO_4_, 5.2 g/L KH_2_PO_4_, 20 g/L glucose, 0.88 g/L trisodium citrate dihydrate, 0.022 g/L ferric ammonium citrate, 2.5 g/L potassium aspartate, 10 mM MgSO_4_, 150 nM MnCl_2_, 40 mg/L L-tryptophan, 0.05% yeast extract), then grown in a 125 mL flask at 37 °C with shaking (250 rpm) until cells reached OD_600_∼1.5. 120 μL of culture were then mixed with up to 10 μL of DNA and incubated at 37 °C with shaking. After 2 hr of incubation, cells were plated on LB agar containing selective antibiotics.

When necessary, the kanamycin resistance cassette flanked by *lox* sequences was removed using Cre recombinase as previously described.^3^ Briefly, a strain containing the *lox*-flanked kanamycin resistance cassette was transformed with pDR244 (a temperature-sensitive plasmid with constitutively expressed Cre recombinase). Transformants were selected on LB agar plates supplemented with 100 μg/mL spectinomycin at 30 °C. Transformants were then streaked on LB agar plates and incubated at 45 °C. Cells from the edge of single colonies were then restreaked on LB, LB supplemented with kanamycin, and LB supplemented with spectinomycin. Strains that grew on LB agar plates but not on LB agar plates supplemented with antibiotics had lost pDR244 and the *lox-*flanked kanamycin resistance cassette.

#### Construction of a modified dCas9 expressing strain

BKC30001 was constructed by replacing the erythromycin-resistance gene of our previously described *dcas9* strain CAG74209^8^ with a fragment containing a kanamycin-resistance cassette flanked with *lox* sites that was generated by joining three PCR fragments: the kanamycin resistance cassette and 1 kb of the 5’- and 3’-flanking regions of the erythromycin-resistance gene in CAG74209. The kanamycin resistance cassette in pDR240a was amplified using primers oDCi005 and oDCi006. 1 kb of the 5’- and 3’-flanking regions of the erythromycin-resistance gene in CAG74209 were amplified using the oDCi001/0DCi002 primer pair and the oDCi003/oDCi004 primer pair, respectively. Amplified DNA fragments were purified using Agencourt AMPure XP magnetic beads. The purified DNA fragments were mixed and subjected to the joining PCR using primers oDCi003 and oDCI006. The joined PCR product was transformed into CAG74209. Three kanamycin-resistant and erythromycin-sensitive clones were isolated and their genomic DNA was purified using the Qiagen DNeasy Blood & Tissue kit. The sequence of *dcas9* was verified by Sanger sequencing (Table S6). The confirmed genomic DNA was re-transformed into the wild-type 168 strain, generating BKC30001.

#### Construction of double sgRNA plasmids

The double sgRNA plasmid pBsuDCi was modified from pDG1662. The pool of pBsuDCi was constructed through three main steps.

First, to increase transformation and double-crossover efficiency, 1.5 kb of DNA upstream of *amyE* was PCR amplified from *B. subtilis* 168 genomic DNA and inserted into pDG1662 via HiFi Assembly (all enzymes and reaction kits used in cloning were purchased from NEB, and high-fidelity versions of restriction enzymes were used if available), replacing the shorter upstream fragment of *amyE* in pDG1662. The synthetic DNA (IDT) containing a transcription terminator, P*veg* with BbsI and P*scr* with BsaI cut sites for spacer cloning, a random barcode sequence, and downstream tandem transcription terminators was cloned into the previously described pDG1662 derivative via HiFi Assembly. The annealed oligonucleotide-containing sgRNA sequence targeting *yabE* with flanking restriction sites was ligated with the purified plasmid digested with BbsI, generating pBsuSCi0.

Second, using pBsuSCi0 as a template, the fragments containing sgRNA1 (Figure 1A; Table S1) and associated random barcodes were individually generated by PCR using the primer pairs of a sgRNA-specific oligonucleotide, oDCi_sgRNA1 and a random barcode-containing oligonucleotide, oDCi014 (Table S6). Each purified fragment was digested with BsrGI and EagI, and cloned into pBsuSCi0, which was digested with the same enzyme followed by dephosphorylation, individually generating a library of pBsuSCi plasmids containing sgRNA1 and a barcode. Barcodes associated with each sgRNA1 were then identified via Sanger sequencing of purified plasmids. The purified equimolar plasmids were pooled in 7 tubes, each of which contained 45∼50 sgRNAs.

Finally, sgRNA2 (Figure 1A; Table S1) was cloned into the BsaI sites of pooled double sgRNA plasmids (pBsuSCi) that contained cloned sgRNA1. sgRNA2 fragments were prepared in two ways. One fraction of sgRNAs was prepared by individually annealing two single-stranded DNA oligonucleotides to create 4-base overhangs, followed by pooling. The rest of the sgRNAs were prepared via digestion of pooled sgRNA fragments with BsaI. To generate sgRNA fragment pools, oligonucleotide pools containing the sgRNA spacers with flanking restriction sites and PCR adapters were obtained from Agilent Technologies. The oligonucleotide pools were amplified via 14 cycles of PCR using Q5 DNA polymerase and primers. The purified PCR product was digested with BsaI-HFv2 and purified after PAGE in 10% TBE gels (Invitrogen) to remove adapter ends. Seven pBsuSCi plasmid pools were individually digested with BsaI-HFv2 for 2 hr. Final double sgRNA plasmid libraries were constructed in two ways depending on the inserts. For the inserts prepared by annealing, the equimolar digested vector pools were combined and ligated with the inserts. In contrast, for the inserts prepared by digestion with BsaI, each vector pool was dephosphorylated and ligated with inserts individually. Each ligation was carried out using 100 ng of digested vector at a 1:2 (vector:insert) molar ratio for 3 hr at 16 °C using T4 DNA ligase. Each of the 8 ligated products was transformed into electrocompetent *E. coli* cells, and cells were recovered in SOC medium at 37 °C for 1 hr, then inoculated into 100 mL of LB with carbenicillin and grown overnight. Each plasmid library was purified using a Qiagen Plasmid Midi kit.

#### Construction of the *B. subtilis* double-CRISPRi library

The double CRISPRi library was constructed by transforming double sgRNA plasmid libraries into BKC30001 using natural competence. The 8 pools of plasmids were linearized via NdeI digestion before transformation to eliminate single-crossover recombination. To increase the transformation scale, the protocol was modified as follows. 300 ng of digested plasmid DNA were mixed with 120 μL of fresh competent cells and incubated in deep 96-well plates. After incubation at 37 °C for 2 hr with shaking (900 rpm), 10 reactions were combined in Eppendorf tubes, and cells were spun down at 5000*g* for 1 min. After discarding 900 μL of supernatant from each tube and resuspending cells, cells were plated on LB agar plates supplemented with chloramphenicol to select for plasmid integration, and the plates were incubated at 37 °C for 16 hr. The yield of each batch of transformation was calculated from CFU counting after serial dilution. The average plating density was ∼3×10^5^ CFUs/plate and the total number of transformants was >100 times the library size. To store the library, plates were scraped, pelleted, and resuspended in S7 salts with 12.5% glycerol, and stored in 500 μL aliquots at −80 °C. The number of clones in each aliquot was calculated by measuring the OD_600_ of the aliquot after serial dilution.

#### Fitness experiments and preparation of Illumina sequencing libraries

Growth experiments with the *B. subtilis* double-CRISPRi library were performed in triplicate and samples were taken as described in Figure S2. Glycerol stocks of 8 pools of the library were fully thawed and inoculated into 500 mL of LB at an OD_600_ of 0.04. These cultures were grown to an OD_600_ of 0.32 at which point all cultures were combined to evenly distribute all clones in one tube. This culture was set as the T0 time point sample. The T0 culture was diluted to an OD_600_ of 0.01 in1 L of fresh LB + 1% xylose and then repeatedly grown to an OD_600_ of 0.32 (∼5 doublings) followed by dilution to an OD_600_ of 0.01 a total of 3 times (to enable 15 doublings), resulting in samples T1, T2, and T3. For the overnight growth and recovery screen, the T0 culture was diluted to an OD_600_ of 0.01 in 1 L of LB (samples T4 and T5) or LB + 1% xylose (samples T6 and T7) and then grown for 18 hr. Each overnight culture (T4 and T6) was diluted to an OD_600_ of 0.01 in 1 L of fresh LB + 1% xylose and then grown to an OD_600_ of 0.32 (∼5 doublings, samples T5 and T7). 1 mL of the culture volume was collected immediately before dilution and after the final growth phase. Cells were pelleted by spinning down at 15,000*g* for 2 min in Eppendorf tubes and stored at −80 °C.

Genomic DNA of the cell pellets was purified using a Qiagen DNeasy Blood & Tissue kit. The sequencing region was amplified from 2 μg of genomic DNA (1000X coverage of each clone) using Q5 DNA polymerase for 14 cycles with primers harboring distinct indices for different replicates and sampling times (Table S6). Differentially indexed PCR products were purified after PAGE in 8% TBE gels and combined at an equimolar ratio. The combined sample was split into three lanes for sequencing on a Novaseq 6000 with 100 bp paired-end reads at the UCSF Center for Advanced Technology using custom sequencing primers (Table S6).

#### Whole-genome sequencing of secondary suppressor strains

Secondary suppressor strains were obtained via transformation of a *mbl*::*kan* fragment into suppressor deletion strains. Many *mbl-*suppressor double-deletion strains, as well as the triple-deletion strains harboring Δ*sigI*, lysed after overnight growth. Secondary suppressor strains regrew from lysed colonies (Note S2) and were purified by picking cells from the regrown colonies followed by restreaking on a fresh LB agar plate. Purified single colonies were grown to an OD_600_ of 1 in LB. 1 mL of each culture was pelleted by spinning down at 15,000*g* for 2 min in Eppendorf tubes. Genomic DNA of the cell pellets was purified using the Qiagen DNeasy Blood & Tissue kit. Purified DNA was submitted to Seqcenter (Pittsburgh, PA, USA) for Illumina 2×151 paired-end sequencing to identify the mutations.

#### High-throughput imaging

Cells from frozen stocks were diluted 1:30 into 300 μL of LB in a deep 96-well plate (Beckman Coulter, cat# 267007), covered with a breathable film, and incubated at 37 °C with shaking at 1000 rpm. After 3 hr of incubation, the culture was diluted to OD_600_∼0.01 in LB with 1% xylose to induce knockdown of the target gene(s) or without xylose and further incubated in a 96-well flat-bottom plate (Greiner Bio-One, Cat# 655180) at 37 °C with shaking at 1000 rpm. After 3 hr of incubation, the culture was passaged again in LB with or without 1% xylose and further incubated in a 96-well flat-bottom plate at 37 °C with shaking at 1000 rpm. OD_600_ was measured using a Biotek Epoch plate reader to monitor growth during the two passages after the initial inoculation. Cells were then transferred from 96-well plates to 1% agar pads with 0.85X PBS using a 96-pin array (Singer Instruments, Cat# REP-001) and imaged using SLIP, a previously described high-throughput single-cell imaging protocol.^81^ Phase-contrast images were acquired with a Ti-E inverted epifluorescence microscope (Nikon Instruments) using a 100X (NA 1.40) oil immersion objective and a Neo 5.5 sCMOS camera (An-dor Technology). Images were acquired using μManager v. 1.4.^83^

#### Cell staining and imaging

After growing the cells in LB with or without induction, they were transferred to an LB agarose pad containing 1% agarose. FM4–64 and DAPI were added directly to the agarose pad at final concentrations of 5 μg/mL and 1 μg/mL, respectively. The cells were then imaged using a Nikon Ti-E inverted epifluorescence microscope equipped with a 100X (NA 1.40) oil immersion objective and a Prime BSI Express sCMOS camera (Teledyne Photometrics). To track fluorescently labeled FtsZ, cells were monitored over several doublings on an LB agarose pad without staining.

#### Microscopy image analysis

Phase-contrast and fluorescence images were analyzed using the MatLab software *Morphometrics*.^84^ A local mesh grid was generated for each cell contour using a method adapted from *MicrobeTracker*^85^ to obtain cell length and width. For each cell, length was determined as the distance along the centerline between the two poles. Lysed cells and cells with fluorescent foci or specific shape defects were manually counted to estimate the frequency of lysis/shape defect in certain mutants. Fluorescently labeled FtsZ rings, when present, were identified as peaks in fluorescence intensity along the cell length and tracked across image frames to determine their lifespan.

### QUANTIFICATION AND STATISTICAL ANALYSIS

#### Relative fitness (RF) quantification

Raw FASTQ files were aligned to the library oligos and enumerated using the script at https://github.com/traeki/mismatch_crispri/blob/master/count_guide_pairs_2021.py, pseudocounts of 1 were added, and relative fitness was calculated as previously described.^26^ Briefly, for each strain *x* with at least 100 counts at *t*_0_, we calculate the relative fitness *F*(*x*) according to

$$F\left(x\right)=\frac{{log}_{2}\left(\frac{r_{wt}(t_{0})r_{x}(t_{10})}{r_{wt}(t_{10})r_{x}(t_{0})}\right)}{g_{wt}}+1$$

where *r*_*x*_(*t*_*i*_) is the fraction of strain *x* in the population after *i* doublings and *g*_wt_ is the number of generations of wild-type growth in the experiment. In our experiments, *g*_wt_ was calculated from the OD measurements of the culture, and *r*_wt_(*t*_*i*_) was calculated as the median of 2024 strains with non-targeting sgRNAs at both positions.

#### GI score calculation and filtering

##### Calculating expected fitness

We used an additive model to calculate expected fitness. The fitness defects (1-RF) of each parent strain were added together and subtracted from 1:

$${RF}_{expected}=1-\left(\left(1-{RF}_{strain\;1}\right)+\left(1-{RF}_{strain\;2}\right)\right).$$

An additive model was chosen over a multiplicative model^9^ for two reasons. First, an additive model makes reasonable predictions if one or both parent strains has negative RF. For example, if RF_parent A_ = −0.5 (i.e., the strain is diluted from the pool faster than dilution, for example via lysis) and RF_parent B_ = 0.5, a multiplicative model would give an expected RF for the double mutant of −0.25, which is less sick than parent A, an illogical conclusion. The situation is even worse if both parent strains have a negative fitness: the expected fitness would then be positive. Second, for the most frequently encountered fitness defects (1 > RF > 0.75), an additive and multiplicative model give similar results. Consider RF_parent A_ = 0.9 and RF_parent B_ = 0.9. The additive definition predicts an expected RF for the double mutant of 0.8, while the multiplicative definition gives 0.81. Our choice is supported by the literature^9^ and by a concurrent study (Dénéréaz et al., co-submitted^78^).

##### GI score calculation

We used custom R code (https://github.com/horiatodor/GI-Score) to determine GI scores. We first identified a set of control strains. To do so, we considered the median across all rows (sgRNA1) and all columns (sgRNA2). ‘‘Control’’ columns were those with column medians within 1 median absolute distance (MAD, a robust analog of standard deviation) of the median of column medians, and ‘‘control’’ rows were those with row medians within 1 MAD of the median of row medians. For each double-CRISPRi strain we then calculated a distribution of expected RF values by adding the fitness defect of all ‘‘control’’ rows and all ‘‘control’’ columns. The GI score was then calculated as the robust (median, MAD) *z*-score of the measured strain fitness. GI scores were calculated separately for the 316×333 library and the 316×982 library since these libraries were constructed separately. GI scores were independently calculated for each of three biological replicates and averaged.

##### Filtering

During the course of our analysis, we found that several sgRNAs had many GIs and that these GIs were correlated with each other. Since these GIs appeared to be due to a systemic artifact, we searched for a technical explanation that would allow us to filter these sgRNAs from the dataset. We found that the GIs of these sgRNAs were highly correlated to the PAM-distal sequence of the interacting sgRNA, which is suggestive of an issue with sgRNA transcription (these bases serve as the transcription start site). We eliminated these spurious hits as follows. For each sgRNA in position 1 (rows), we performed a linear regression between its GI scores and the first 2 nucleotides of the interacting sgRNA. The first 2 nucleotides were one-hot encoded to convert these categorical variables into a matrix of binary values that can be used in linear regression, as previously described.^26^ We constructed a distribution of correlations, and eliminated all sgRNAs with a correlation greater than the median plus 5 times the MAD of the distribution of correlations. The same process was applied to sgRNAs at position 2 (columns). Approximately 10% of strains were removed through this process.

##### Correlation matrix calculation

The correlation of GI scores was calculated as the Pearson correlation between all sgRNAs at position 2 (columns), resulting in a 1315 × 1315 matrix.

##### STRING analysis

Interactions from the STRING database were retrieved from https://string-db.org for *Bacillus subtilis* strain 168 (taxid: 224308). The composite channel, which integrates information from all channels (neighborhood, fusion and co-occurrence, co-expression, experiments, database, text mining) from the STRING database, was used.

## Supplementary Material

MMC5

MMC3

MMC1

MMC6

MMC7

MMC2

MMC9

MMC8

MMC4

Supplemental information can be found online at https://doi.org/10.1016/j.cels.2025.101406.

## Figures and Tables

**Table:** KEY RESOURCES TABLE

REAGENT or RESOURCE	SOURCE	IDENTIFIER
Chemicals, Peptides, and Recombinant Proteins		
Lysogeny broth (LB), Lennox	Fisher scientific	Cat# BP1427-2
Agar	BD Biosciences	Cat# 214030
Yeast extract	BD Biosciences	Cat# 212750
Casamino acids	BD Biosciences	Cat# 223050
Potassium phosphate monobasic	Sigma-Aldrich	Cat# P0662
Potassium phosphate dibasic	Sigma-Aldrich	Cat# P8281
Trisodium citrate dihydrate	Fisher scientific	Cat# S279-500
Ferric ammonium citrate	Sigma-Aldrich	Cat# F5879
Potassium glutamate monohydrate	Sigma-Aldrich	Cat# G1501
Potassium aspartate	Sigma-Aldrich	Cat# A6558
Magnesium sulfate heptahydrate	Sigma-Aldrich	Cat# M1880
Manganese chloride tetrahydrate	Sigma-Aldrich	Cat# M3634
L-tryptophan	Fisher scientific	Cat# BP395-100
Dextrose (D-[+]-glucose)	Sigma-Aldrich	Cat# D9434
Glycerol	Fisher scientific	Cat# G30-4
MOPS	Sigma-Aldrich	Cat# M1254
Potassium sulfate	Sigma-Aldrich	Cat# P9458
Ammonium chloride	Sigma-Aldrich	Cat# A9434
Kanamycin sulfate	Sigma-Aldrich	Cat# K1377
Erythromycin	Sigma-Aldrich	Cat# E5389
Lincomycin hydrochloride	Sigma-Aldrich	Cat# L2774
Spectinomycin dihydrochloride pentahydrate	Sigma-Aldrich	Cat# S9007
Ampicillin sodium salt	Sigma-Aldrich	Cat# A9518
Carbenicillin disodium salt	Sigma-Aldrich	Cat# C3416
Xylose (D-[+]-xylose)	Sigma-Aldrich	Cat# X1500
Starch, soluble	Sigma-Aldrich	Cat# S9765
Lysozyme (from chicken egg white)	Sigma-Aldrich	Cat# L6876
Triton X-100	Fisher scientific	Cat# BP151-500
Q5 High-Fidelity DNA polymerase	New England Biolabs	Cat# M0491L
dNTP solution mix	New England Biolabs	Cat# N0447
T4 DNA ligase	New England Biolabs	Cat# M0202
BbsI-HF	New England Biolabs	Cat# R3539
BsaI-HFv2	New England Biolabs	Cat# R3733
BsrGI-HF	New England Biolabs	Cat# R3575
EagI-HF	New England Biolabs	Cat# R3505
NdeI	New England Biolabs	Cat# R0111
NEBNext Q5 Hot Start HiFi PCR Master Mix	New England Biolabs	Cat# M0543
OneTaq Hot Start Quick-Load 2X Master Mix	New England Biolabs	Cat# M0488
10% TBE PAGE gel	Invitrogen	Cat# EC6275BOX
8% TBE PAGE gel	Invitrogen	Cat# EC6215BOX
FM4-64	Invitrogen	Cat# T13320
DAPI	Sigma-Aldrich	Cat# D9542
Agarose	Invitrogen	Cat# 16500500
Quick-Load Purple1kb Plus DNA Ladder	New England Biolabs	Cat# N0550
50bp DNA Ladder	New England Biolabs	Cat# N3236
Critical Commercial Assays		
Agencourt AMPure XP	Beckman Coulter	Cat# A63881
QIAprep Spin miniprep kit	Qiagen	Cat# 27106
QIAquick PCR purification kit	Qiagen	Cat# 28104
DNeasy Blood & Tissue kit	Qiagen	Cat# 69506
Plasmid Midi kit	Qiagen	Cat# 12143
NEBuilder HiFi DNA Assembly Cloning kit	New England Biolabs	Cat# E5520S
NEB 10-beta Competent *E. coli*	New England Biolabs	Cat# C3019
NEB 10-beta Electrocompetent *E. coli*	New England Biolabs	Cat# C3020
Deposited Data		
Raw FASTQ files for relative fitness experiments and whole genome analysis		NCBI SRA -Bioproject: PRJNA1143566
Experimental Models: Organisms/Strains		
Strains used in this study are listed in Table S5.		
Recombinant DNA		
Plasmids used in this study are listed in Table S5.		
Sequence-Based Reagents		
Primers used in this study are listed in Table S6.		
Software and Algorithms		
sgRNA design (fully matched sgRNAs)	Hawkins et al.^26^	https://github.com/traeki/sgrna_design/blob/master/shuffle_sgrna_library.py; https://doi.org/10.5281/zenodo.16740690
Design a subset of mismatch sgRNA (choose_guides.py)	Hawkins et al.^26^	https://github.com/traeki/mismatch_crispri; https://doi.org/10.5281/zenodo.16740688
FASTQ analysis to calculate sgRNA abundance	This study	https://github.com/traeki/mismatch_crispri/blob/master/count_guide_pairs_2021.py; https://doi.org/10.5281/zenodo.16740688
Calculation of GI score	This study	https://github.com/horiatodor/GI-Score; https://doi.org/10.5281/zenodo.16701406
μManager v. 1.4	Edelstein et al.^83^	N/A
Morphometrics	Ursell et al.^84^	N/A
MicrobeTracker	Sliusarenko et al.^85^	N/A

## Highlights

- Double-CRISPRi maps genetic interactions in *Bacillus subtilis* at the genome scale
- Correlated genetic interaction profiles reveal pathway connections
- Different roles for actin homologs in peptidoglycan and teichoic acid synthesis
- New genes linked to cell division expand the envelope biogenesis network
