# Supplementary material for: Comprehensive genetic interaction analysis of the Bacillus subtilis envelope using double-CRISPRi
Source: Cell Syst. Author manuscript; Available in PMC 2025 Dec 19. (PMC12716459; doi:10.1016/j.cels.2025.101406)
Supplement: MMC1 [file NIHMS2115161-supplement-MMC1.pdf]

Cell Systems, Volume *16*

Supplemental information

Comprehensive genetic interaction analysis of the *Bacillus subtilis* envelope using double-CRISPRi

Byoung-Mo Koo, Horia Todor, Jiawei Sun, Jordi van Gestel, John S. Hawkins, Cameron C. Hearne, Amy B. Banta, Kerwyn Casey Huang, Jason M. Peters, and Carol A. Gross

Document S1

Supplemental Figures S1 ~ S11

Supplemental Notes 1~3, including Figures S12, S13, and Tables S8~S10

Supplemental References

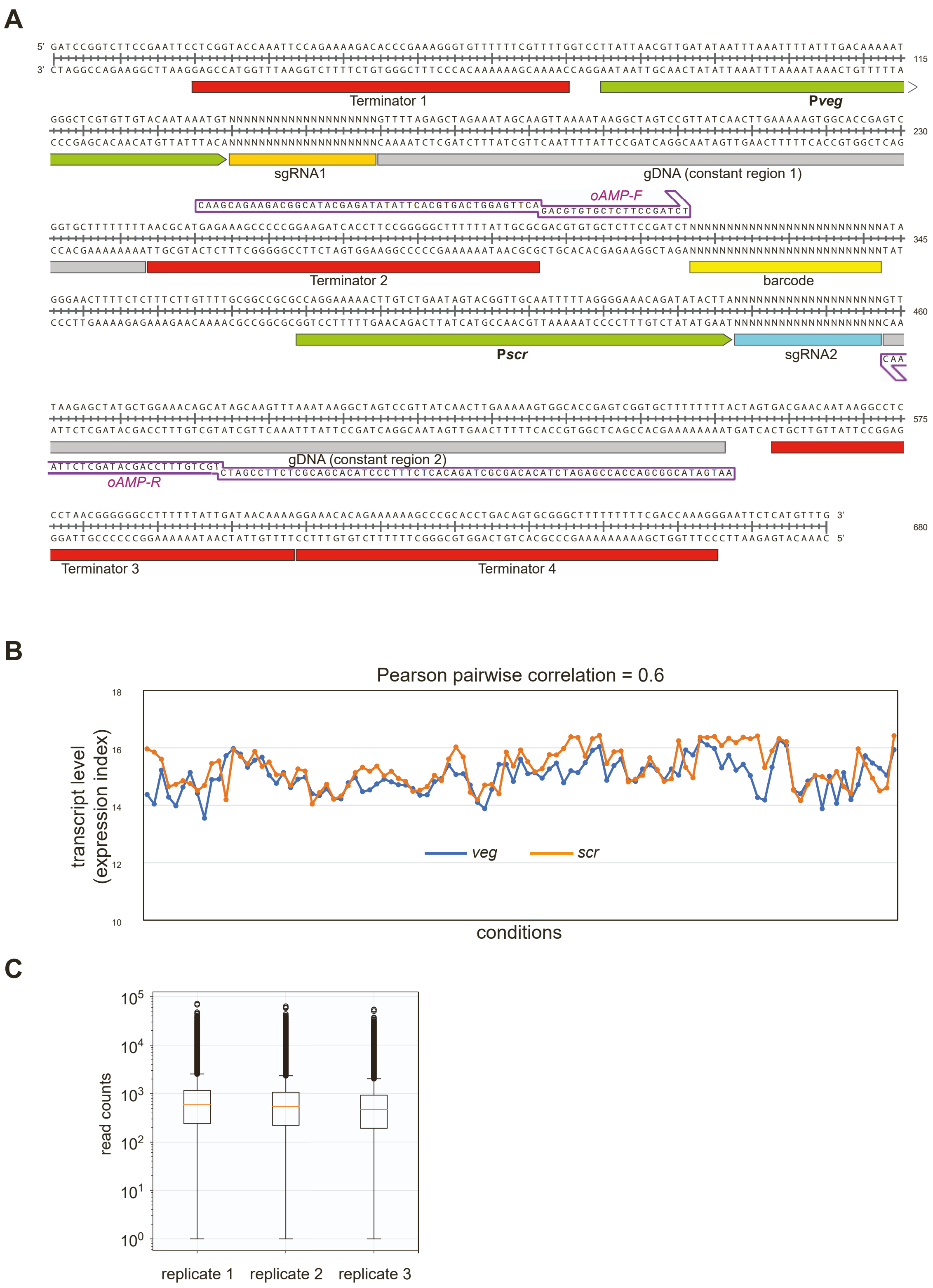

Figure S1. Chromosomally-integrated double-CRISPRi system in *B. subtilis*, related to Figure 1

A) Detailed nucleotide sequence of sgRNA locus. Each genetic feature is indicated under sequence.

B) Transcriptional profiles of veg and scr whose promoters were used for transcription of sgRNA1 and sgRNA2 respectively. Data was obtained from SubtiWiki[1].

C) Distribution of read counts of sgRNA pairs in the three replicates of T0 samples (Figure S2). Distribution is represented by a box plot. Orange line indicates the median of the read counts (replicate 1; 592, replicate 2; 546, replicate 3; 469)

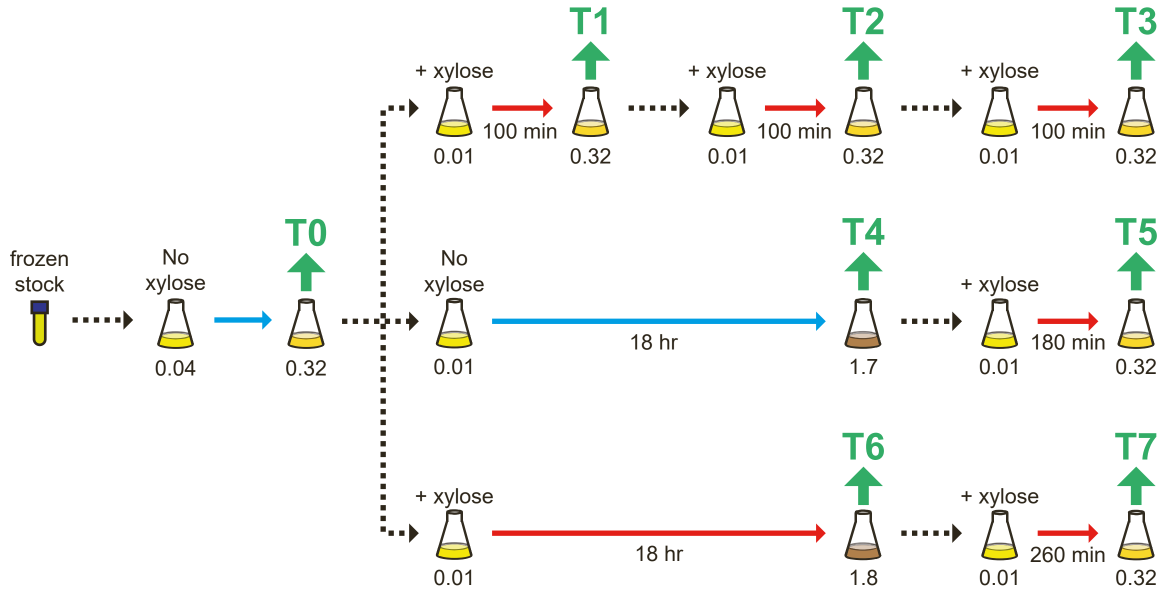

Figure S2. Schematic of the pooled growth experiment and sampling time points, related to Figure 1
Straight blue arrows represent growth without xylose (CRISPRi system OFF). Straight red arrows represent growth with 1% xylose (CRISPRi system ON). Broken lines represent back dilution. OD600 are denoted below each flask. Green arrows represent timepoints at which cells were collected (T0-T7). Samples were collected at these time points to determine the optimal generation times for the GI screen during exponential growth and to assess GI during recovery from overnight culture. The interpretation for all pairwise comparisons is described in the “preface” sheet in the Table S2, S3, and S4 in addition to Table S7. For each timepoint, replicates were obtained from the cultures of three independent 1liter flasks.

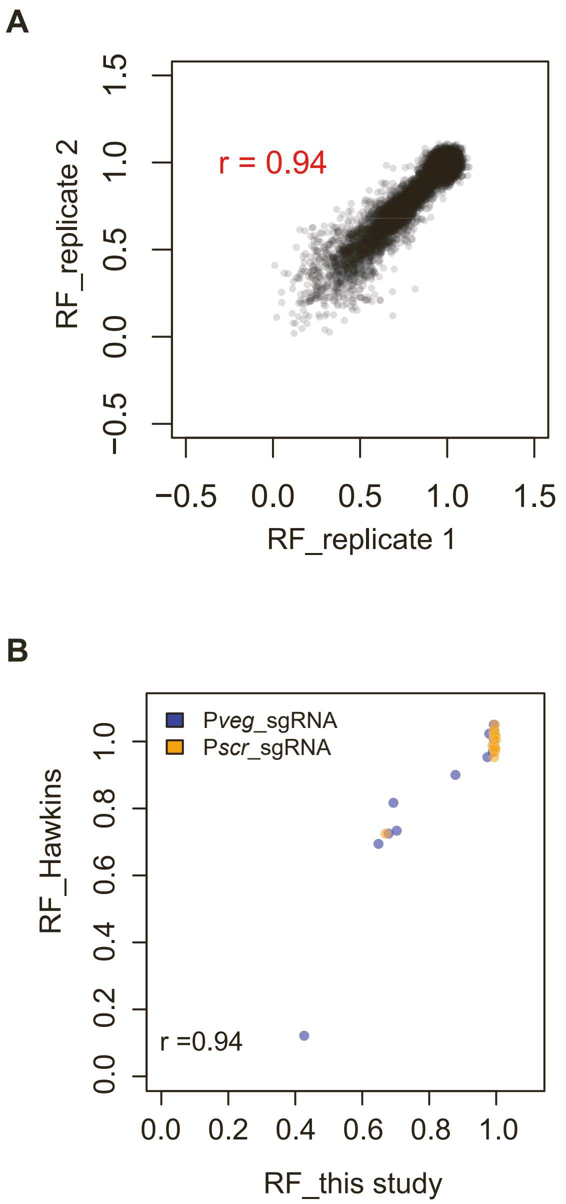

Figure S3. The RFs of the strains obtained in this study are highly reproducible, related to Figure 1
A) Correlation between RF of three replicates.
B) Correlation between RF of single gene knockdown strains in this study and the relevant strains in previously published single-CRISPRi experiments[2]. Blue and orange dots indicate knockdown induced by sgRNA under P*veg* and P*scr* respectively.

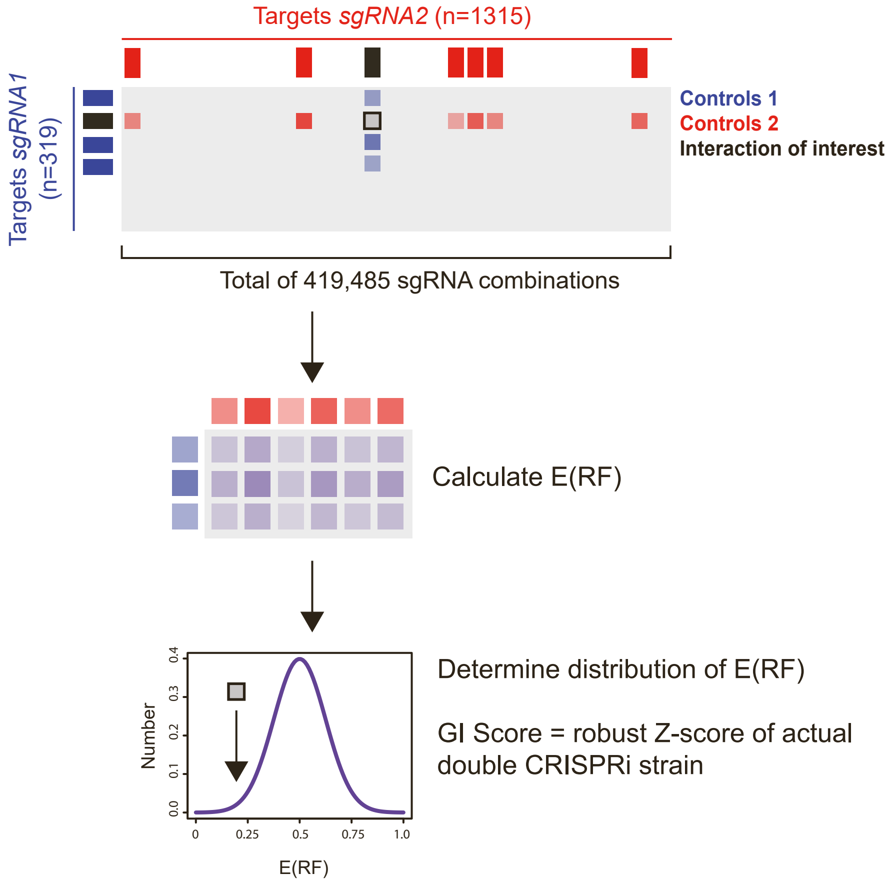

Figure S4. Calculation of GI scores, related to Figure 2
See Methods.

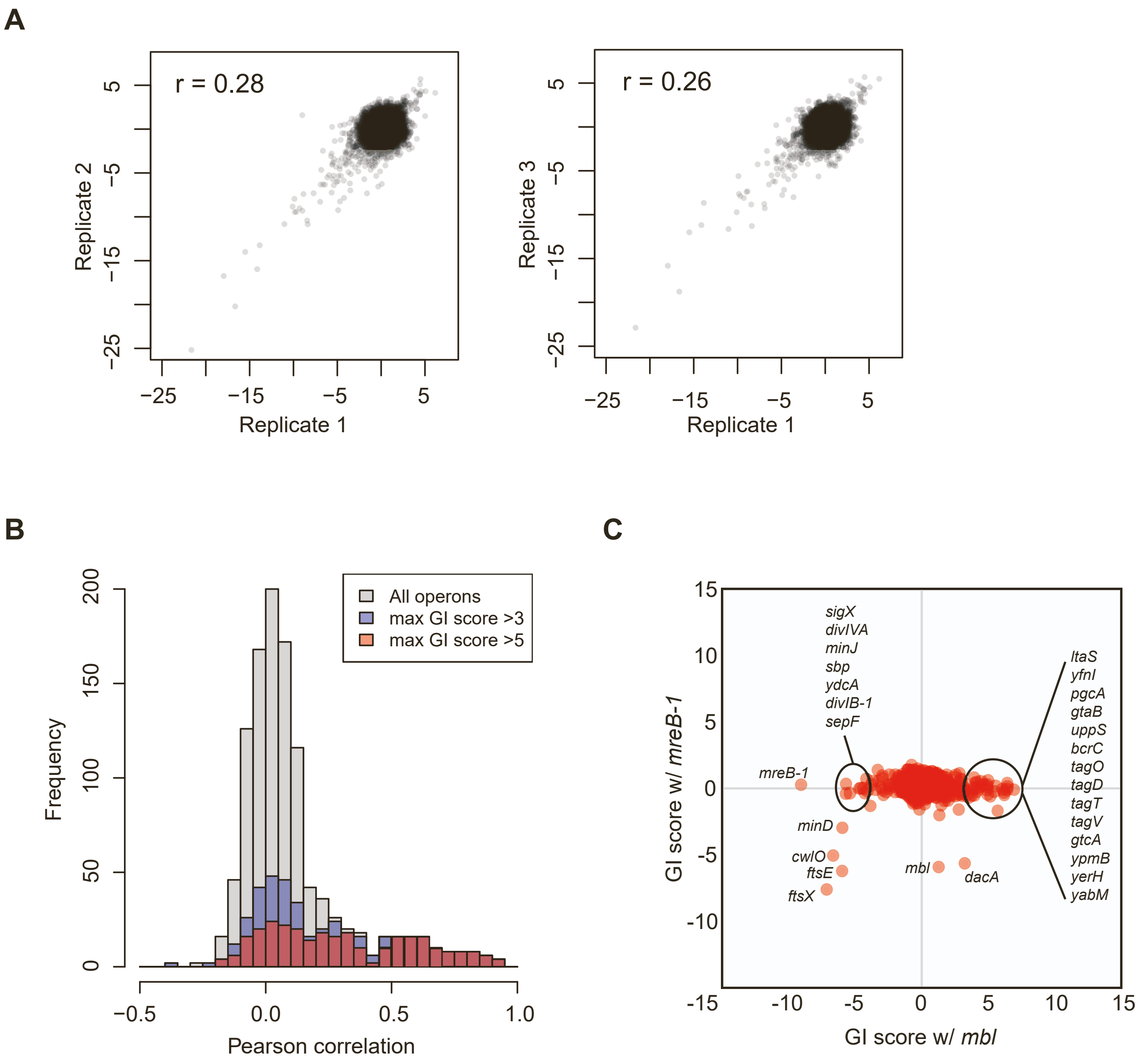

Figure S5. Double-CRISPRi identifies GIs, related to Figure 2

A) Correlation between all GI scores of three replicates. Note that correlations are higher with a high GI score threshold. (r ~ 0.79 for at least one replicate with |GI-score|>3; r ~ 0.59 for at least one replicate with |GI-score|>2).
B) Distribution of the GI correlations between the genes within the same operon.
C) GI scores for all strains with *mbl* (x-axis) and *mreB* (y-axis).

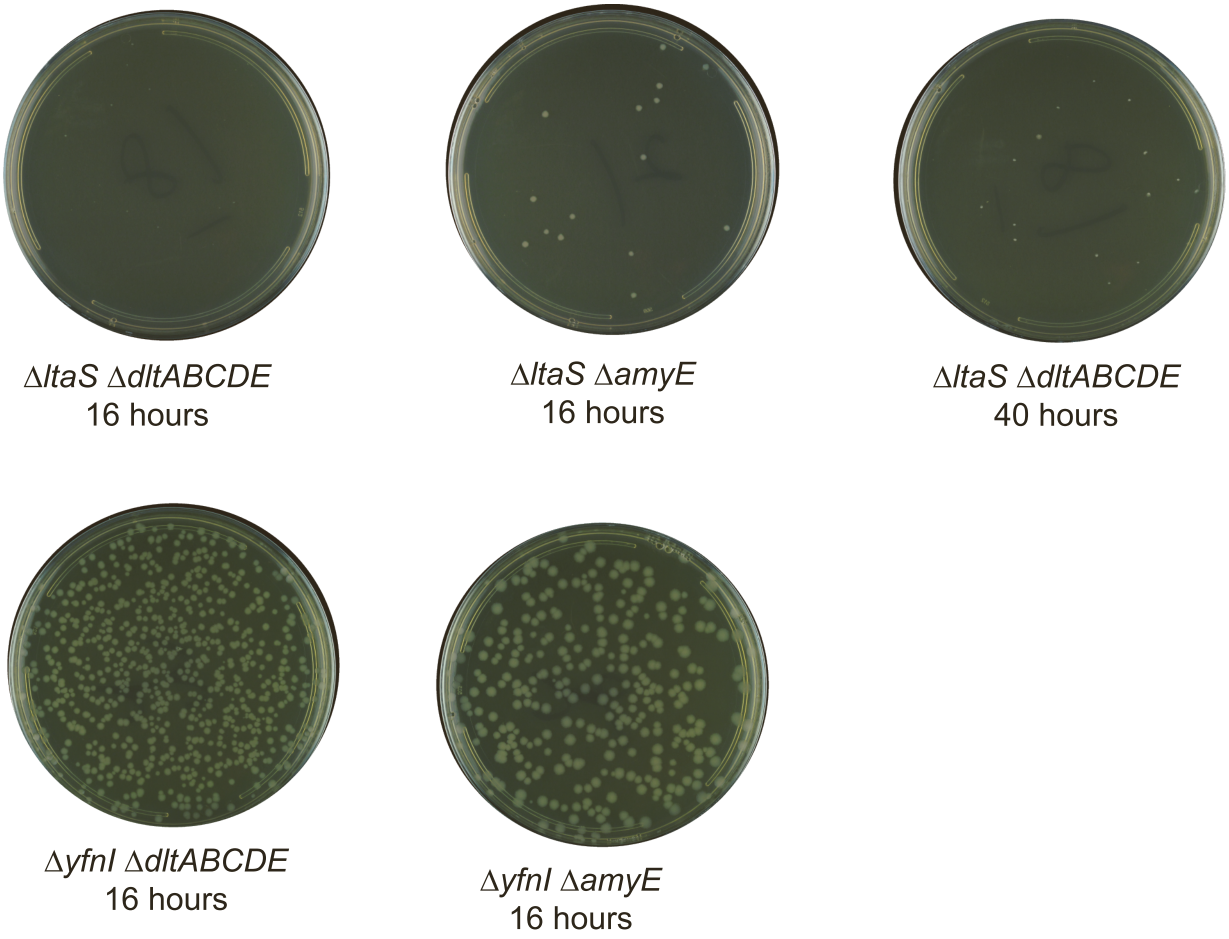

Figure S6. The defect in D-alanylation significantly impairs the growth of *ΔltaS* strain but not that of *ΔyfnI* strain, related to Figure 4

dlt operon deletion (*dltABCDE::kan*) and *amyE::kan* fragments (as a transformation control) were transformed into antibiotic marker-free *ΔltaS* strain and *ΔyfnI* and incubated for 16 hours. Colonies of *ΔltaS ΔdltABCDE* double mutant were not visible on the plate after 16 hours of incubation but were visible after 40 hours.

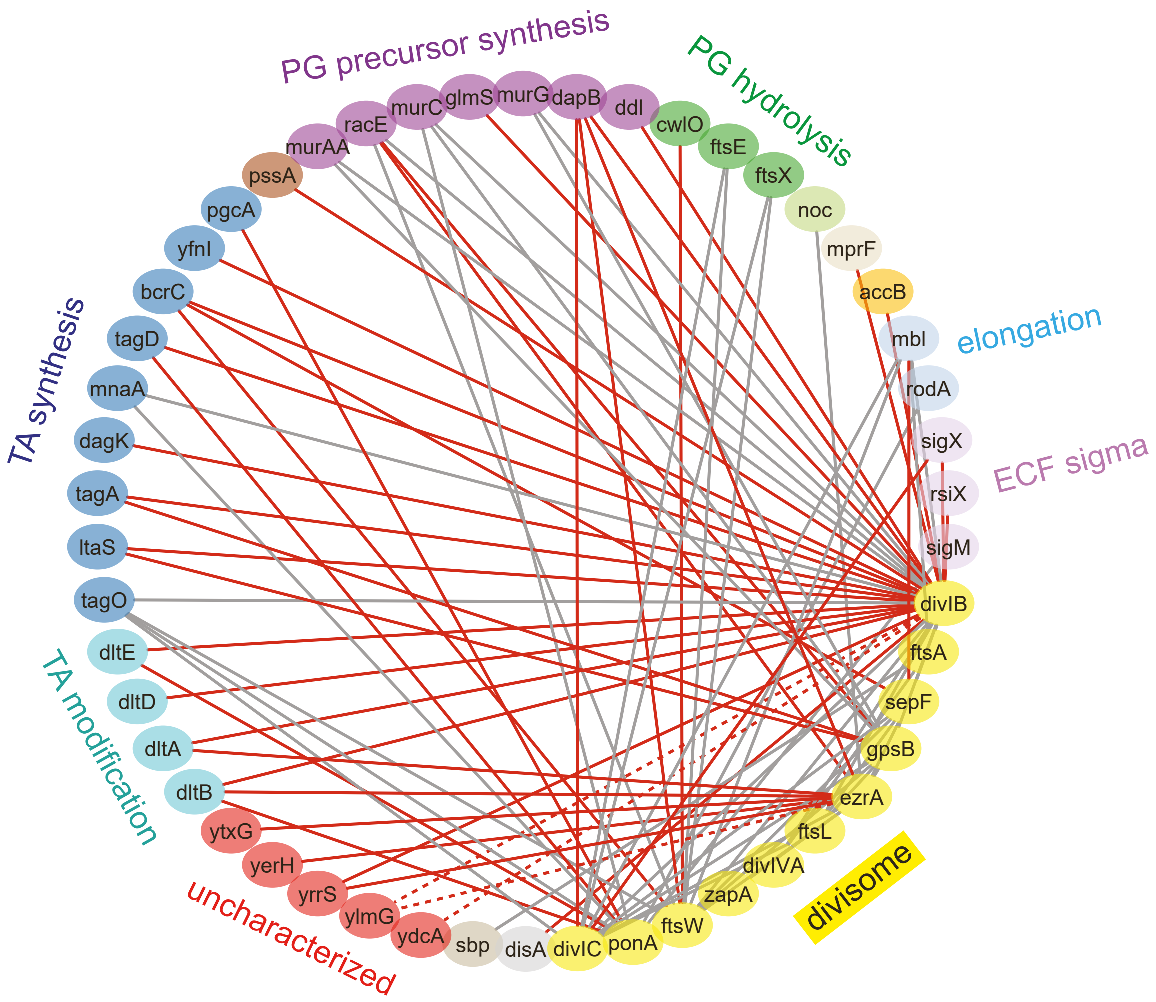

Figure S7. A detailed GI network of divisome genes, related to Figure 6
The network is identical to Figure 6B. Gray lines indicate known interactions and red lines indicate novel interactions identified in this screen.

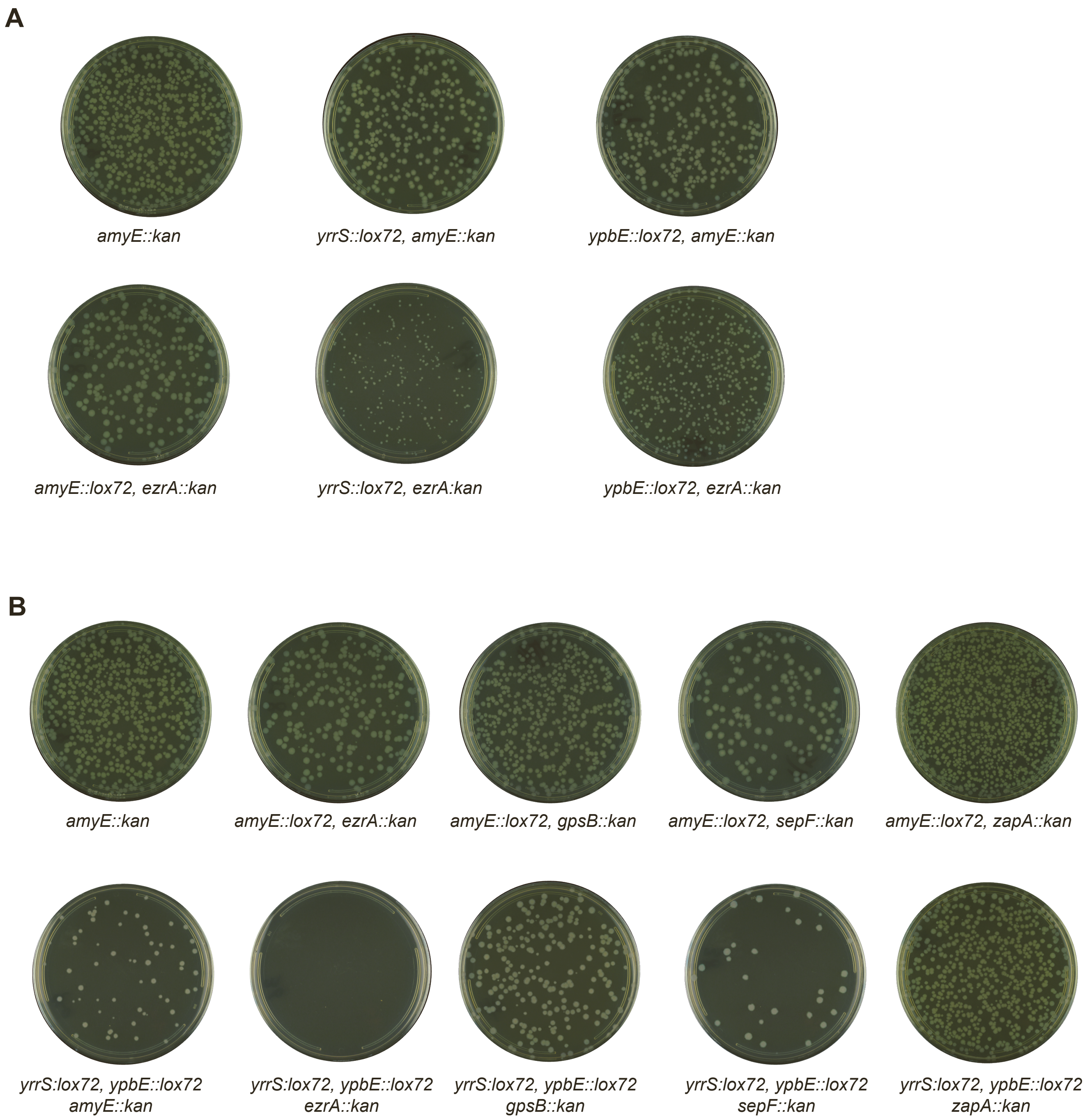

Figure S8. Deletion of *yrrS* and *ypbE* exhibit synthetic growth phenotype with deletion of *ezrA*, but not with those of other *ezrA*-interacting cell division genes such as *gpsB*, *sepF*, and *zapA*, related to Figure 6

A) Growth phenotypes of Δ*yrrS*Δ*ezrA* and Δ*ypbE*Δ*ezrA*.

B) Growth phenotypes of triple mutants. Colonies of Δ*yrrS*Δ*ypbE*Δ*ezrA* triple mutant were not visible on the plate after 16 hours of incubation but were visible after 40 hours.

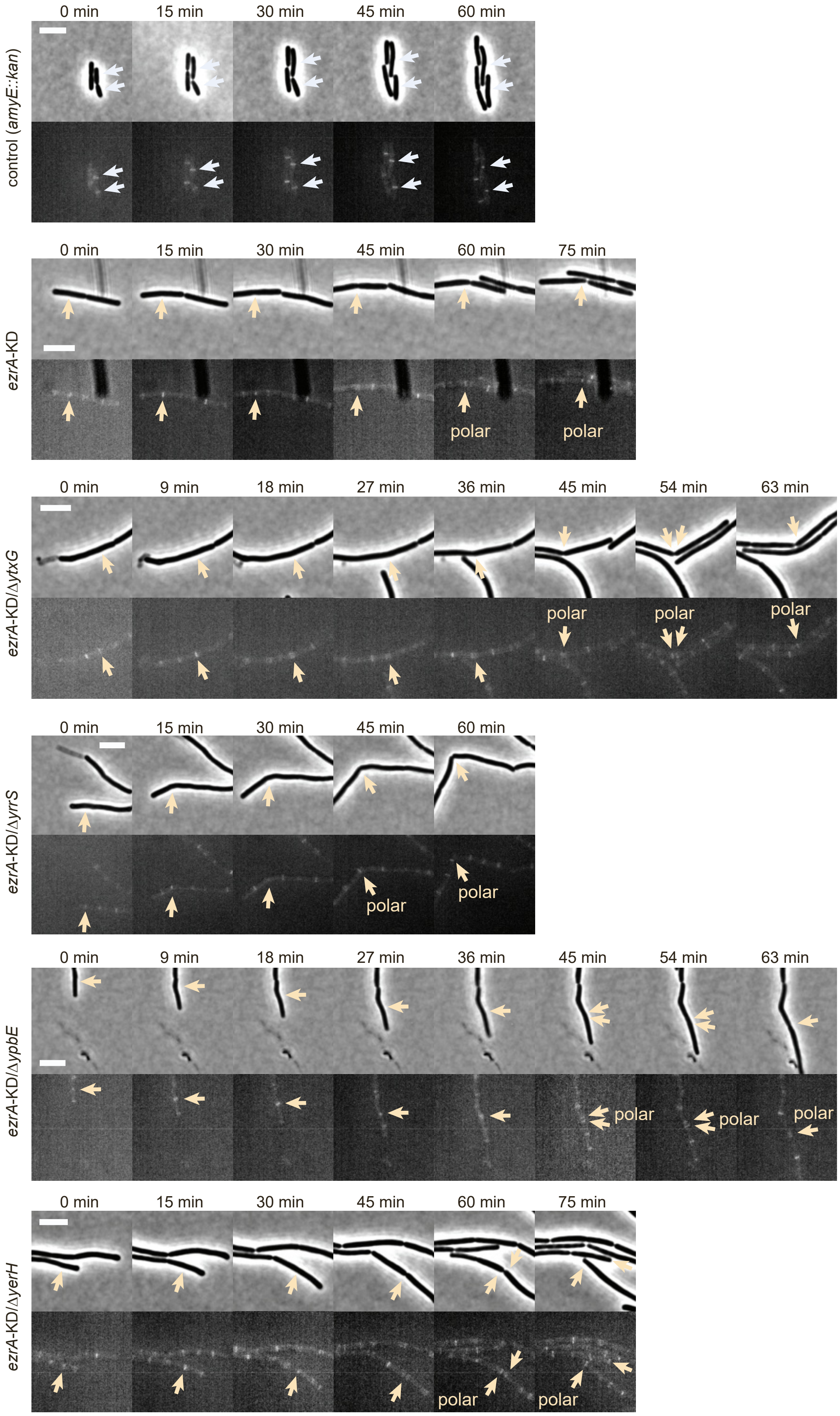

control (amyE::kan)
0 min
15 min
30 min
45 min
60 min
ezrA-KD
0 min
15 min
30 min
45 min
60 min
75 min
polar
polar
ezrA-KD/ΔytxG
0 min
9 min
18 min
27 min
36 min
45 min
54 min
63 min
polar
polar
polar
ezrA-KD/ΔyrrS
0 min
15 min
30 min
45 min
60 min
polar
polar
ezrA-KD/ΔypbE
0 min
9 min
18 min
27 min
36 min
45 min
54 min
63 min
polar
polar
polar
ezrA-KD/ΔyerH
0 min
15 min
30 min
45 min
60 min
75 min
polar
polar

Figure S9. *ezrA* depletion alone or with Δ*yrrS*, Δ*ypbE*, Δ*ytxG*, or Δ*yerH* exhibited persistent Z-rings at the adjacent new poles after cell division, related to Figure 7

For each mutant, individual cells were imaged using phase-contrast microscopy (top row) and NeonGreen fluorescence (bottom row) microscopy. Arrows indicate Z-rings of interest, with polar Z-rings specifically labeled. Scale bars: 5 μm.

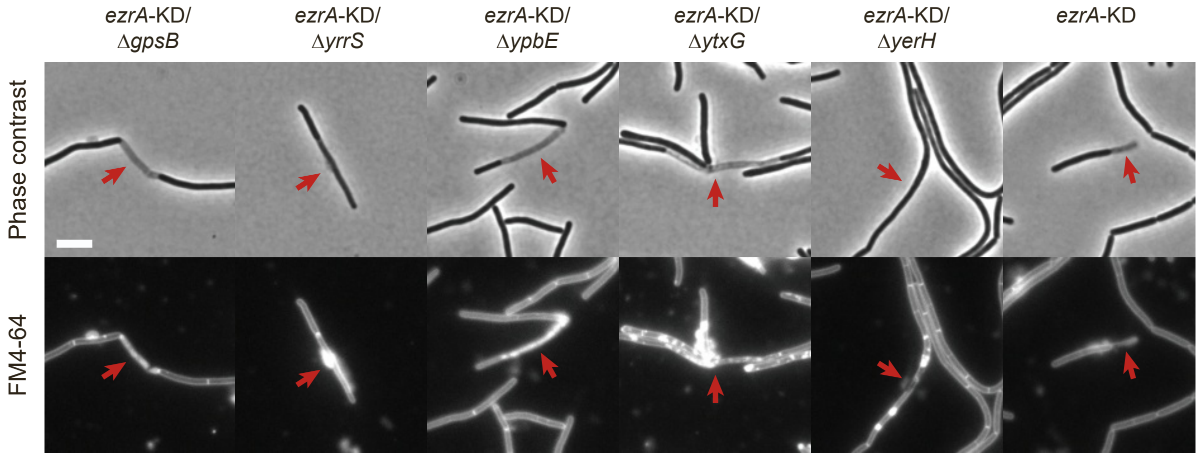

Figure S10. Lysis of cells within a filament does not affect neighboring compartments, indicating that their cytoplasm is fully separated, related to Figure 7

Arrows indicate lysed cells. Scale bar: 5 µm.

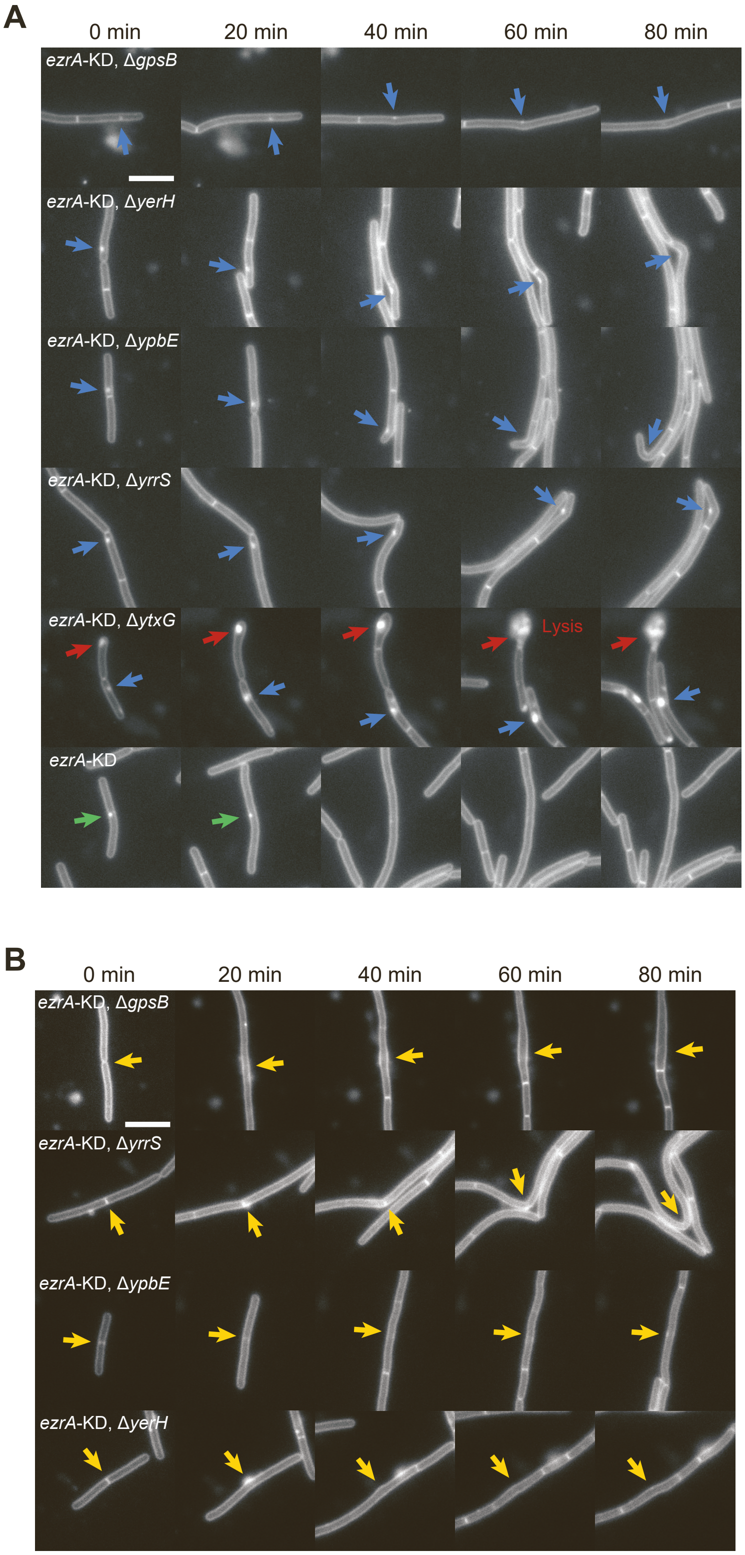

Figure S11. Knockout mutants of several uncharacterized genes exhibit distinct shapes and membrane phenotypes under *ezrA* depletion, related to Figure 7

A) Cells with bright FM4-64 foci along the cell body can develop sharp kinks due to asymmetric cell wall growth (blue arrows). *ΔytxG* cells with bright patches around cell poles can exhibit bulging poles and/or lysis (red arrows). Without additional gene knockouts, bright foci under *ezrA* depletion typically does not cause shape defects. Scale bar: 5 µm.

B) Examples of cell poles or FM4-64 cross-bands that disappear after formation (yellow arrows). In some cases, swelling (*ΔgpsB*) or bending (*ΔyrrS* and *ΔyerH*) occurs at the location of the disappearing pole/cross-band during subsequent growth of the cell wall. Arrows indicate the poles/cross-bands or the subsequent shape defect. Scale bar: 5 µm.

Supplemental Note 1, related to Figure 3

GI profile of *ydcA*, an uncharacterized gene

In our screen *ydcA* GIs were highly correlated with those of the *dlt* operon and *sigX* (Figure 3C and Table S8). *ydcA* has strong negative GIs with *pgcA*, *gtaB*, *divIB*, *mbl*, *prsW*, *cwlO*, and *ggaA*. As expected, these negative GIs (except for *mbl* and *prsW)* were shared with the *dlt* operon and *sigX* (Table S9). To better understand these interactions, we constructed two pairs of double deletion mutants (*pgcA/ydcA* and *gtaB/ydcA*) using pairwise donor-recipient combinations in transformations (*pgcA::lox72/ydcA::kan*, *ydcA::lox72/pgcA::kan*, *gtaB::lox72/ydcA::kan*, *ydcA::lox72/gtaB::kan*). Interestingly, only the mutants harboring the kanamycin-resistance gene replacing *ydcA* recapitulated the negative GIs, suggesting that the kanamycin-resistance gene at *ydcA* rather than the *ydcA* deletion itself was the major contributor to the observed phenotypes.

This finding prompted us to examine the genomic context of the *ydcA* gene locus. We found that the almost the entirety of the *ydcA* gene overlapped the 5'UTR of *acpS*, which encodes the essential acyl-carrier protein synthetase and is transcribed in the opposite direction from *ydcA* (Figure S12). Given this genomic arrangement, two explanations for the GIs of *ydcA* are possible. First and most likely, CRISPRi knockdown of *ydcA* or replacement of *ydcA* with the kanamycin-resistant gene may reduce the expression of *acpS* by targeting the template strand of its promoter with CRISPRi or by producing antisense RNA for 5'UTR of *acpS* via strong transcription of the kanamycin-resistance gene. AcpS interacts with DltC, and this interaction is essential for D-alanylation of TA[3,4]. This implies that *acpS* knockdown partially produces the phenotype of *dlt* genes knockdown. Consistent with the idea that the phenotypes of *ydcA* are due to downregulation of *acpS*, *ydcA* is a member of SigE regulon and therefore primarily expressed during sporulation rather than under normal growth conditions such as LB[5]. Together, these data suggest that the phenotypes of the *ydcA* knockdown strain result from the partial knockdown of *acpS* followed by reduced D-alanylation of TA. A second, though less likely, explanation for the phenotype is that reduced expression of *ydcA* itself produced GI with other genes. A *ydcA* homolog in *S. aureus*, *actH* was identified

as an activator of LytH, an amidase involved in cell growth and division[6]. Thus, *ydcA* may contribute to cell wall homeostasis by modulating certain cell wall hydrolase activity even though we didn't observe any phenotype for *yqiI*, a homolog of S. aureus *lytH*.

Table S8. Highly correlated GI with *ydcA* (Pearson's r >0.5)

gene	correlation with *ydcA*
sigX	0.84
dltE	0.73
dltA	0.69
dltB	0.68
dltD	0.67
rsiX	0.57

Table S9. GI profile of *ydcA* (|GI|>2.5) and highly correlated genes

sgRNA	GI_*ydcA*	GI_*sigX*	GI_*dltE*	GI_*dltA*	GI_*dltB*	GI_*dltD*	GI_*rsiX*
pgcA	-10.23	-10.84			-5.70	-5.44	
gtaB	-10.11	-10.43	-10.74	-7.86	-6.89	-3.90	
divIB-1	-9.07	-8.80	-3.01	-3.60	-3.66	-3.37	-5.61
divIB-2	-6.61	-9.92	-4.98	-5.81	-4.09	-5.27	
mbl	-4.54	-5.71	-2.65	-0.80	-0.84	-0.74	
prsW	-2.84	0.24	-1.19	0.90	0.11	0.45	
cwlO	-2.80	-2.65	-4.51	-3.17	-1.96	-3.48	-2.51
ggaA	-2.72	-3.01	-4.46	-3.06	-2.92	-3.85	-1.13

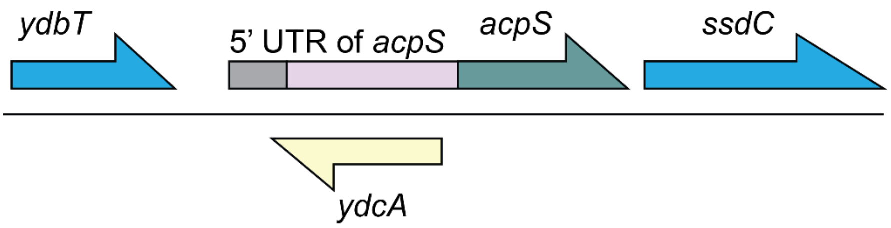

Figure S12. Genomic context of *ydcA* locus

Supplemental Note 2, related to Figure 5

Further characterization of the Δ*mbl*-suppressor mutants

Activation of the sigma factor SigI (and presumably upregulation of its operon) by deletion of the gene encoding its anti-sigma factor *rsgI* was previously shown to suppress the essentiality of *mbl*[7]. We therefore tested whether the *mbl* suppressors we isolated worked by activating SigI. Importantly, we were able to reconstruct many *mbl*-suppressor double-deletions in a Δ*sigI* background (Table S10). The triple mutant growth phenotypes were similar to those of the double mutants, suggesting that these suppressive mechanisms are SigI-independent.

We observed that many of our *mbl*-suppressor double-deletion strains, as well as the triple-deletion strains harboring Δ*sigI* lysed after overnight growth. This observation is consistent with the less positive GI scores of the *mbl*-suppressor strains following overnight growth observed in the double-CRISPRi screen (Table S3), and was also apparent on LB agar plates as lysed colonies after extended growth (Figures S13A and S13B). To identify mutations that further stabilize the *mbl*-suppressor strains, we isolated 11 colonies that outgrew from lysed colonies (Figure S13C) and sequenced their genomes. Suppressors isolated in both the double- and triple-deletion strains mapped to: *walK,* the kinase component of the essential cell wall homeostasis regulator WalRK; its negative regulator *walH*; and *yhdK*, the negative regulator of the cell wall sigma factor SigM (Table S10)[8,9]. Mutations and truncations in negative regulators are commonly loss-of-function mutants. Therefore the observed mutations likely increase WalK sensor kinase activity or activate SigM, which is required for the regulation of PG synthesis. Thus, the suppressors identified in our screen partially compensate for the lack of Mbl during exponential growth, but further activation of PG synthesis systems is required for survival during stationary phase growth.

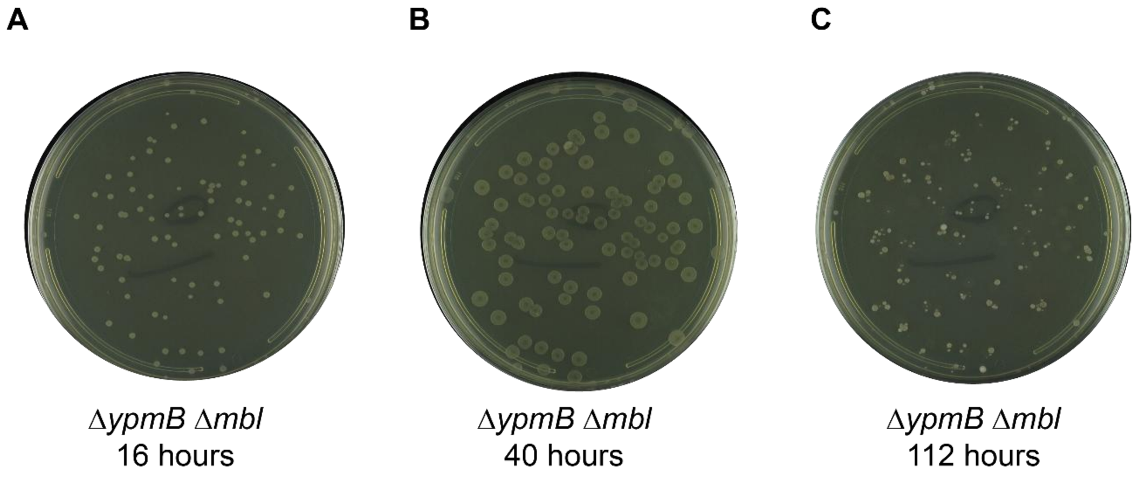

Figure S13. The growth of mbl and suppressor double deletion mutant. *mbl::kan* fragment was transformed into an antibiotic marker-free *ypmB* deletion strain and incubated for A) 16 hours, B) 40 hours, and C) 112 hours. We observed similar growth patterns of most of the other *mbl*/suppressor double deletion mutants on the LB agar plates.

Table S10. List of the second mutations that might stabilize mbl/suppressor double deletion strains.

Strain	genotype	second mutations		
		mutation/position	gene	predicted phenotype
BKS0010	*ltaS::lox72, mbl::kan*	L190V (TTG→GTG)	*walK*	WalK activation?
BKS0020	*bcrC::lox72, mbl::kan*	Δ14 bp after 837/1368 nt	*walH*	WalK activation
BKS0030	*ypmB::lox72, mbl::kan*	P391S (CCG→TCG)	*walK*	WalK activation?
BKS0040	*ypmB::lox72, mbl::kan*	Δ2 bp after 655/1368 nt	*walH*	WalK activation
BKS0050	*yvcK::lox72, mbl::kan*	L198F (CTC→TTC)	*walK*	WalK activation?
BKS0060	*yvcK::lox72, mbl::kan*	S143N (AGT→AAT)	*sigM*	SigM activation?
BKS0070	*sigI::lox72, yfnI::lox72, mbl::kan*	Δ21 bp after131/291 nt	*yhdK*	SigM activation
BKS0080	*sigI::lox72, yfnI::lox72, mbl::kan*	V553G (GTG→GGG)	*walK*	WalK activation?
BKS0090	*sigI::lox72, yvcK::lox72, mbl::erm*	Δ1 bp after 721/1368 nt	*walH*	WalK activation
BKS0100	*sigI::lox72, yvcK::lox72, mbl::erm*	F244S (TTT→TCT)	*walK*	WalK activation?
BKS0110	*sigI::lox72, yerH::lox72, mbl::kan*	duplication (CGGCAGCCATGGCG AACGGAA) at 131/291 nt	*yhdK*	SigM activation

Supplemental Note 3, related to Figure 6

Additional GI data support a role for *yrrS*, *ypbE, ytxG*, and *yerH* in cell division

Our newly identified cell division genes all exhibited negative GIs with *ezrA*, but also had distinct GI, as evidenced by the lack of significant GI correlations among them (Table S3 and S4).

Like *ezrA* and *gpsB,* both *yrrS* and *ytxG* exhibited negative GIs with *ponA* (encodes PBP1), raising the possibility that they, like EzrA and GpsB, play a role in PBP1 localization. As YpbE physically interacts with YrrS and has a similar protein-protein interaction profile to that of YrrS[10], it may also be involved in this process. These proteins may have additional roles in division as there are no significant GI correlations among them (Table S4). For example, *yrrS* has a strong negative GI with *divIB* whereas *ytxG* has many negative GIs with other genes including *mreB, uppS, and bcrC*. Interestingly, as mentioned above, *yerH* suppressed the *mbl* phenotype, suggesting that this gene might be involved in a connection between cell elongation and division independent of GpsB. In summary, our GI data and morphological analysis data support the idea that we have identified new players in cell division.

Supplemental References

1. Pedreira, T., Elfmann, C., and Stulke, J. (2022). The current state of SubtiWiki, the database for the model organism Bacillus subtilis. Nucleic Acids Res *50*, D875-D882. 10.1093/nar/gkab943.
2. Hawkins, J.S., Silvis, M.R., Koo, B.M., Peters, J.M., Osadnik, H., Jost, M., Hearne, C.C., Weissman, J.S., Todor, H., and Gross, C.A. (2020). Mismatch-CRISPRi Reveals the Co-varying Expression-Fitness Relationships of Essential Genes in Escherichia coli and Bacillus subtilis. Cell Syst *11*, 523-535 e529. 10.1016/j.cels.2020.09.009.
3. Ma, D., Wang, Z., Merrikh, C.N., Lang, K.S., Lu, P., Li, X., Merrikh, H., Rao, Z., and Xu, W. (2018). Crystal structure of a membrane-bound O-acyltransferase. Nature *562*, 286-290. 10.1038/s41586-018-0568-2.
4. Nikolopoulos, N., Matos, R.C., Courtin, P., Ayala, I., Akherraz, H., Simorre, J.P., Chapot-Chartier, M.P., Leulier, F., Ravaud, S., and Grangeasse, C. (2022). DltC acts as an interaction hub for AcpS, DltA and DltB in the teichoic acid D-alanylation pathway of Lactiplantibacillus plantarum. Sci Rep *12*, 13133. 10.1038/s41598-022-17434-2.
5. Eichenberger, P., Jensen, S.T., Conlon, E.M., van Ooij, C., Silvaggi, J., Gonzalez-Pastor, J.E., Fujita, M., Ben-Yehuda, S., Stragier, P., Liu, J.S., and Losick, R. (2003). The sigmaE regulon and the identification of additional sporulation genes in Bacillus subtilis. J Mol Biol *327*, 945-972. 10.1016/s0022-2836(03)00205-5.
6. Do, T., Schaefer, K., Santiago, A.G., Coe, K.A., Fernandes, P.B., Kahne, D., Pinho, M.G., and Walker, S. (2020). Staphylococcus aureus cell growth and division are regulated by an amidase that trims peptides from uncrosslinked peptidoglycan. Nat Microbiol *5*, 291-303. 10.1038/s41564-019-0632-1.
7. Schirner, K., and Errington, J. (2009). The cell wall regulator sigmaI specifically suppresses the lethal phenotype of mbl mutants in Bacillus subtilis. J Bacteriol *191*, 1404-1413. 10.1128/JB.01497-08.
8. Szurmant, H., Nelson, K., Kim, E.J., Perego, M., and Hoch, J.A. (2005). YycH regulates the activity of the essential YycFG two-component system in Bacillus subtilis. J Bacteriol *187*, 5419-5426. 10.1128/JB.187.15.5419-5426.2005.
9. Zhao, H., Roistacher, D.M., and Helmann, J.D. (2019). Deciphering the essentiality and function of the anti-sigma(M) factors in Bacillus subtilis. Mol Microbiol *112*, 482-497. 10.1111/mmi.14216.
10. Cleverley, R.M., Rutter, Z.J., Rismondo, J., Corona, F., Tsui, H.T., Alatawi, F.A., Daniel, R.A., Halbedel, S., Massidda, O., Winkler, M.E., and Lewis, R.J. (2019). The cell cycle regulator GpsB functions as cytosolic adaptor for multiple cell wall enzymes. Nature communications *10*, 261. 10.1038/s41467-018-08056-2.
