# Supplementary material for: Comprehensive genetic interaction analysis of the Bacillus subtilis envelope using double-CRISPRi
Source: Cell Syst. Author manuscript; Available in PMC 2025 Dec 19. (PMC12716459; doi:10.1016/j.cels.2025.101406)
Supplement: MMC9 [file NIHMS2115161-supplement-MMC9.pdf]

Cell Systems

Transparent Peer Review Record

# Comprehensive genetic interaction analysis of the Bacillus subtilis envelope using double-CRISPRi

Byoung-Mo Koo, Horia Todor, Jiawei Sun, Jordi van Gestel, John S. Hawkins, Cameron C. Hearne, Amy B. Banta, Kerwyn Casey Huang, Jason M. Peters, Carol A. Gross

## Summary

Initial Submission: Received September 3, 2024
Preprint: doi.org/10.1101/2024.08.14.608006
Scientific editor: Bernadett Gaal, DPhil

First round of review: Number of reviewers: 3
*3 confidential, 0 signed*
Revision invited Jan 14, 2025
*Major changes anticipated*
Revision received June 12, 2025

Second round of review: Number of reviewers: 2
*2 original, 0 new*
*2 confidential, 0 signed*
Accepted Aug 28, 2025

*This Transparent Peer Review Record is not systematically proofread, type-set, or edited. Special characters, formatting, and equations may fail to render properly. Standard procedural text within the editor's letters has been deleted for the sake of brevity, but all official correspondence specific to the manuscript has been preserved.*

## Editorial decision letter with reviewers' comments, first round of review

Dear Dr. Gross,

I'm enclosing the comments that reviewers made on your paper, which I hope you will find useful and constructive. As you'll see, they express interest in the study, but they also have a number of criticisms and suggestions. Based on these comments, it seems premature to proceed with the paper in its current form; however, if it's possible to address the concerns raised with additional experiments and/or analysis, we'd be interested in considering a revised version of the manuscript.

I'm always happy to talk, either over email or by phone, if you'd like feedback about whether your efforts are moving the manuscript in a productive direction. Do note that we generally consider papers through only one major round of revision, so the revised manuscript would be either accepted or rejected based on the next round of comments we receive from the reviewers. If you have any questions or concerns, please let me know. More technical information and advice about resubmission can be found below my signature. Please read it carefully, as it can save substantial time and effort later.

**STAR PROTOCOLS**

Consider complementing your primary research article by publishing a step-by-step procedure with STAR Protocols, an open-access peer-reviewed journal from Cell Press. STAR Protocols aims to make the daily work of the scientific researcher easier by providing complete, authoritative, and consistent instructions on how to conduct experiments. The primary criteria for publication in STAR Protocols is usability and reproducibility. You can check out their most recent protocols here. If you have any questions, please email starprotocols@cell.com.

I look forward to seeing your revised manuscript.

All the best,

Bernadett

Bernadett Gaál, DPhil
Editor-in-Chief, Cell Systems

### Reviewers' comments:

Reviewer #1: The double CRISPRi study from Koo et al. presents a large-scale analysis of genetic interactions (GIs) for genes previously implicated in envelope homeostasis in the Gram-positive model organism B. subtilis. The results cover the interactions between 333 well characterized genes (mostly known envelope genes) and 982 poorly characterized membrane-localized or envelope-associated genes. Well over 10E5 interactions are quantified as a relative fitness (growth rate) score. It is very difficult to determine what is new here vs. what is confirmatory (much is in the latter category). This work, more carefully prepared, might represent a useful for systems biologists to explore as a hypothesis-generating tool, and the authors have provided some initial thoughts on GIs that are probably worth further exploration. Efforts should be made to more clearly document how the

dataset was used to identify novel interactions, so that readers wishing to make use of this resource will have a clear model to follow.

Comments
1. Overall comment: several sections of the text need to be explained better. It is hard in places to see precisely how the authors arrive at their conclusions, or even what comparisons are being made.
2. Title: "Comprehensive double-mutant analysis…." Are these really double mutants? The genes are repressed by CRISPRi, not deleted or mutated.
3. Abstract: The authors state that their work will "reveal the distinct roles of paralogous genes such as the mreB and mbl." It is possible to briefly state what these distinct roles are for those readers who might be intrigued?
4. Para from line 117. Can the authors define envelope genes? In infer that they include peptidoglycan and teichoic acid synthesis pathways, known regulators, and later steps in membrane biogenesis. Other genes are missing, and it is not clear why (the acc genes are included, but the fatty acid synthase genes are not? etc.)
5. Line 119. Why not be specific about the number of envelope genes queried (333 includes many controls). To me it looks like 272 genes (?) (media 3, Table S1)
6. Line 129. The value of 1315 used here includes all of the control genes (~45?) from set 1. It might be more useful for readers to state what the actual number of "test" genes are from set 1 vs. set 2 (corrected for those that failed for technical reasons). This number is not easily determined as written.
7. Line 140. The reference to "multiple time points" here is confusing. Does "T" refer to time? Or to transfer? There are seven different transfers shown in Fig S2. It might be worth describing this in the Results section. The logic of the different transfer schemes is not obvious. What comparisons are meant to be made? The SI materials in media 4 and media 5 make several pair-wise comparisons to determine GI scores. Where are these described?
8. Line 175 and line 729. The STRING database will also include "interactions" between genes anytime the gene names appear together in the literature ("text mining"). Was this type of interaction turned off for this analysis? Please the setting used (and rationale).
9. Figure 2D. I do not think there is enough information for readers to find the raw data used, for example, in Figure 2D. Which set of RF data form the basis of this heatmap? What transfers are compared? And why were these pairs chosen? This is generally true for similar figures -- please specify the actual source file for the raw data used to generate the plots!
10. Figure 3C. As above. The basis for this heatmap (in the extensive SI datasets) is not obvious. It would be very helpful for readers to understand how you used the raw data to make these graphs.
11. line 182. Why unexpected? Based on the schematic given and prior work, I would say that these are expected interactions. Perhaps "previously unreported" would be more appropriate.
12. Line 186. Why a novel interaction? The mreB and mbl genes are well known to genetically interact.
13. Section after line 191. A supplemental table of correlated GI profiles would be useful! For example, of the large set of poorly characterized genes, readers may want to see if their favorite gene has a correlated profile with other genes. This work has apparently been done, so why not include the results as a new SI table?
14. Line 220. I do not think the word "paralogous" is justified here. Some of the mentioned gene pairs are paralogs, but I think others are not.
15. Line 286. YdaH has been renamed Amj. The use of current gene names is encouraged, but since all the raw data has BSU# for each gene this is not a major barrier to further analysis.
16. I got lost in Fig. 5. There are candidate suppressors in Fig. 5A, but apparently none of these suppress? A bit more explanation would help to tie the description (in results) to the figure.
17. Line 674. What it "t10" in this equation?

18. Line 702. Define MAD.
19. Line 719. Not clear what "one-hot" means,
20. Line 38: Not exclusively….Much of classical genetics was based on altered function mutations (e.g. temperature sensitive, etc.) in addition to loss of function.
21. Please check grammar in media 1.
22. The SI tables are potentially useful, but the intent of the different comparisons (in the different tabs) is not obvious. Please add text to explain, and make sure the cells do not have errors in them.

Reviewer #2: Overall comments：
Understanding the genetic interactions (GIs) between functional genes can help us decipher their important biological significance. The biological traits reflected by some important genes are still not deciphered, and even these genes are closely related to important research fields such as environmental control and antibiotic resistance.
In this manuscript, the author developed double-CRISPRi technology for genome-scale GI screen of envelope genes in model Gram-positive bacterium Bacillus subtilis. The idea of selecting the envelope process is very nice (as it is closely related to pathogenicity and drug resistance), and the analysis of this process can deepen our understanding of the envelope process of Gram-positive bacteria cells and provide a new paradigm for Gram-positive bacteria. The author used this technology to identify over 1,000 known and novel positive and negative GIs. They also demonstrated that paralogous genes are not functionally similar or redundant, but rather play different roles such as the mreB and mbl actin homologs. In summary, the selected research subjects for this study are excellent, the manuscript is well written, and the results are reliable. However, several issues need to be clarified before this study can be accepted.
Major issues:
1. The expression of dCas9 is induced through the xylose-induced promoter system in this double-CRISPRi system. As far as I know, xylose promoter has certain leakage expression in B. subtilis. Does this affect the conclusion of the experiment?
2. I agree with the author on the fact that repeat sequences are more likely to be lost in B. subtilis because B. subtilis has a rich DNA recombination system. However, repeat sequences (such as sgRNA scaffold) are more prone to recombination and deletion when DNA bends, as designed by the author.
3. Figure 5C: ΔmreB ΔptsI and Δmbl ΔptsI showed significant differences in colony size, suggesting that mreB and mbl appear to be equally important for cell growth (perhaps of different importance or with other genes acting as complements). However, the ΔmreB ΔyfnI mutant showed significant growth inhibition compared to the Δmbl ΔyfnI mutant, where mreB and mbl seemed to play different roles. Perhaps mreB and mbl play different roles under specific conditions (But it's not absolute.), thus, I do not agree that mreB and mbl are "distinct roles" as described in the Abstract.
4. Figure 6C: Using the constructed double mutants, the author found that all four y-genes exhibited negative GIs with ezrA but not gpsB. The author seem to have missed the negative control ΔgpsB ΔezrA.
5. Single-cell imaging (Figure 7B): Lack of control (amyE::Kan), please add.
Minor issues:
1. Figure 6C: Please use the subscript below for OD600
2. Figure 6C: Please change "ΔyterH" to "ΔyerH"
3. Summary: line 26: but it is difficult to implement at the genome-scale.
4. Summary: line 28: We discover > 1000 known to "We discover > 1,000 known"
5. Introduction: line 40: high throughput techniques to "high-throughput techniques"
6. Introduction: line 82: identification of synthetic-lethal gene pairs to "the identification of synthetic-lethal gene pairs"

7. Introduction: line 86: "envelope-function genes necessary to" to "envelope-function genes is necessary to"
8. Results: line 105: Because loss of repeated to "Because the loss of repeated"
9. Results: line 118: select essential genes to "selected essential genes"
10. Results: line 185: data set to "dataset"
11. Results: line 210: highly correlated to that of genes in their regulon to "highly correlated with those of genes in their regulon"
12. Results: line 242: Other GIs with bcrC motivate additional study to "Other GIs with bcrC motivate additional studies"
13. Perspective: line 432: gene function and pathway connections to "gene functions and pathway connections"
14. Perspective: line 459: mis-localization to "mislocalization"
15. Limitations: line 512: yrrS unaffected by pbpI knockdown to "yrrS that is unaffected by pbpI knockdown"

Reviewer #3: The manuscript by Koo & Todor et al deals with the construction and characterization of double-CRISPRi libraries in Bacillus subtilis, with a specific focus on cell envelope genes. On the whole I believe the work presented in the manuscript will be a useful resource for those studying the cell envelope in related bacteria. A strength of the manuscript is that the double-CRISPRi approach was able to recapitulate existing genetic interactions and reveal new ones. Generally speaking however, I found that the manuscripts runs a little short on the functional characterization of the most interesting, novel genetic interactions, particularly if we consider the plethora of cell biology tools available to characterize the bacterial cell envelope. As a systems biology focused manuscript, it does however fulfil the purpose of elucidating existing and novel genetic interactions and providing a novel resource for the community of cell envelope enthusiasts.

Specific comments:

Knockdowns - can the authors present evidence of knockdown looking specifically at the transcript levels for the genes that they chose to focus on in for example in Figure 6 & 7?

Line 139/140 - How did the authors decide on 10 doubling times post-induction?

Figure 6C - growth curves - it seems like the authors only conducted two biological replicates. Why not 3? Would it make more sense to present the doubling time instead?

Figure 7: Why did the authors choose to use phase contrast and then FM4-64 to measure cell length? For the analysis, how did the authors deal with the cells that are visibly stressed? Where these included in the analysis? How many cells were counted? How many biological replicates?

Figure 7: Did the authors also measure the interchromosome (i.e. internucleoid) distance? It seems like several of these mutants (ytxG, yerH) may have a larger interchromosomosal distance? Did the authors also determine the frequency of cell lysis?

Figure S2 - legend - can the authors include more detail in the legend - it would be useful to have the text between line 645 and 658 as the legend for this figure.

## Authors' response to the reviewers' first round comments

Attached.

---

## Editorial decision letter with reviewers' comments, second round of review

Dear Carol,

I hope this email finds you well. The reviews are back on your manuscript and I've appended them below. You will see that while the Reviewers are largely satisfied with your revisions, Reviewer 3 points out that it remains unclear whether the data in Figure 7 come from a single experiment or were pooled from replicates. This will need to be clarified and the reproducibility of these results demonstrated before we can move forward.

If any changes to the authorship are needed (addition, removal, or a change in order of authors), we require that an authorship change form be provided with the revised manuscript that indicates the reason for the change and provides written consent for the change from all authors, including any that were removed.

I look forward to seeing your revised manuscript.

All the best,

Bernadett

Bernadett Gaál, DPhil
Editor-in-Chief, *Cell Systems*

### Reviewer comments:

Reviewer #2: The authors have addressed most of my previous concerns and improved the manuscript accordingly. The revisions are satisfactory, and the paper now meets the journal's standards.

Reviewer #3: The authors have addressed most of my comments. However, the comments about the data in Figure 7A have not been addressed.

The authors state:

In the analysis, we included all cells, both visibly stressed and not. Thus, each statistics in Figure 7A reflects the entire population. We counted n>200 cells for each distribution.

All images were acquired in a single microscopy session, which captured the major phenotypes (cell elongation and cytoplasmic compartmentalization) with enough cells to represent the distribution of cellular dimensions. We have updated the figure caption to include these details.

However, the figure caption has not been updated and this data is not from 3 biological replicates but from a single experiment. How do the authors know these data are representative? It seems like these experiments were done only once and not repeated. Furthermore, the authors include statistics, but they have not mentioned what statistical test was used. This needs to be addressed because it is hard to know if this data is reproducible or not. Furthermore, it is not clear what statistical test was used. The authors need to be demonstrate reproducibility in their work.

## Authors' response to the reviewers' second round comments

Attached.

**Detailed responses to the reviewers' comments**

*Note: Reviewer comments are in bold, author responses are in regular font, and updated manuscript text is shown in blue.*

**Reviewer #1:**

**The double CRISPRi study from Koo et al. presents a large-scale analysis of genetic interactions (GIs) for genes previously implicated in envelope homeostasis in the Gram-positive model organism B. subtilis. The results cover the interactions between 333 well characterized genes (mostly known envelope genes) and 982 poorly characterized membrane-localized or envelope-associated genes. Well over 10E5 interactions are quantified as a relative fitness (growth rate) score. It is very difficult to determine what is new here vs. what is confirmatory (much is in the latter category). This work, more carefully prepared, might represent a useful for systems biologists to explore as a hypothesis-generating tool, and the authors have provided some initial thoughts on GIs that are probably worth further exploration. Efforts should be made to more clearly document how the dataset was used to identify novel interactions, so that readers wishing to make use of this resource will have a clear model to follow.**

**Comments**
**1. Overall comment: several sections of the text need to be explained better. It is hard in places to see precisely how the authors arrive at their conclusions, or even what comparisons are being made.**

We thank the reviewer for drawing our attention to possible ambiguities. To clarify the manuscript, we made a number of changes that are described throughout the response to reviewer comments. Briefly:

- We made changes to the title and abstract as suggested by the reviewer.
- We added details to the text regarding how we chose genes.
- We overhauled the legend for Fig. 5 and Fig. S2 to better support the text.
- We changed the language around paralogs to reflect the reviewer's concerns.
- We made a host of smaller changes to improve readability.
- We added details to the methods section to better explain our experiments, analysis, and assumptions.

We think these changes address the reviewer's concerns and have substantially improved the manuscript.

**2. Title: "Comprehensive double-mutant analysis…." Are these really double mutants? The genes are repressed by CRISPRi, not deleted or mutated.**

In response to the reviewer's point, we have changed the title to:

"Comprehensive genetic interaction analysis of the *Bacillus subtilis* envelope using double-CRISPRi"

**3. Abstract: The authors state that their work will "reveal the distinct roles of paralogous genes such as the mreB and mbl." It is possible to briefly state what these distinct roles are for those readers who might be intrigued?**

We have changed the abstract as suggested by the reviewer. The sentence now reads:

Line 30,
"Our analysis pipeline and experimental follow-ups reveal the shared and distinct roles of paralogous genes such as *mreB* and *mbl* in peptidoglycan and teichoic acid synthesis and identify new genes involved in the well-studied process of cell division."

**4. Para from line 117. Can the authors define envelope genes? In infer that they include peptidoglycan and teichoic acid synthesis pathways, known regulators, and later steps in membrane biogenesis.**

We have changed the text to include more details. It now reads:

Line 114,
"Our screen targeted envelope-function genes, such as those involved in peptidoglycan or teichoic acid synthesis, the later steps of membrane biogenesis, known regulators, and genes of unknown function with predicted transmembrane helices as categorized in SubtiWiki[25]. We focused on interactions between two sets of genes (Figure 1B). The first set consists of sgRNAs targeting well-characterized envelope genes, selected essential genes, and non-targeting controls (333 total; categories 1,2; Figure 1B). Essential genes were targeted by mismatched sgRNAs[26] that produce mild knockdown and moderate growth defects, to enable the quantification of both positive and negative GIs. The second set consists of poorly characterized membrane-localized or envelope-associated genes (982 total; categories 3,4; Figure 1B and Table S1)."

**Other genes are missing, and it is not clear why (the acc genes are included, but the fatty acid synthase genes are not? etc.)**

We did not include most predicted sporulation genes and prophage genes. Some interesting genes are unfortunately missing from the library since the list of genes was selected 10 years ago at the inception of library creation and some genes were not appropriately annotated in SubtiWiki (e.g., *acc* genes). Some genes of specific interest were added.

**5. Line 119. Why not be specific about the number of envelope genes queried (333 includes many controls). To me it looks like 272 genes (?) (media 3, Table S1)**

We apologize for the confusion; we have changed the text to reflect the exact numbers.

**6. Line 129. The value of 1315 used here includes all of the control genes (~45?) from set 1. It might be more useful for readers to state what the actual number of "test" genes are from set 1 vs. set 2 (corrected for those that failed for technical reasons). This number is not easily determined as written.**

We were able to confirm that 316 of 333 genes in set 1 were successfully cloned, since they were cloned individually. However, since sgRNAs in set 2 were cloned as pools, each sgRNA targeting the genes in set 2 was not exhaustively combined with all sgRNAs targeting genes in set 1. Thus, the description of the actual number of "test" genes in set 2 is not informative.

All strains that were constructed and screened for growth are listed in Table S2.

**7. Line 140. The reference to "multiple time points" here is confusing. Does "T" refer to time? Or to transfer? There are seven different transfers shown in Fig S2. It might be worth describing this in the Results section. The logic of the different transfer schemes is not obvious. What comparisons are meant to be made? The SI materials in media 4 and media 5 make several pair-wise comparisons to determine GI scores. Where are these described?**

We have updated and expanded the Figure S2 legend to better explain the figure. It now reads:

"**Figure S2**. Related to Figure 1. **Schematic of the pooled growth experiment and sampling time points.** Straight blue arrows represent growth without xylose (CRISPRi system OFF). Straight red arrows represent growth with 1% xylose (CRISPRi system ON). Broken lines represent back dilution. $OD_{600}$ are denoted below each flask. Green arrows represent timepoints at which cells were collected (T0-T7). Samples were collected at these time points to determine the optimal generation times for the GI screen during exponential growth and to assess GI during recovery from overnight culture. The interpretation for all pairwise comparisons is described in the "preface" sheet in the Table S2, S3, and S4 in addition to Table S7. For each timepoint, replicates were obtained from the cultures of three independent 1liter flasks.
"

**8. Line 175 and line 729. The STRING database will also include "interactions" between genes anytime the gene names appear together in the literature ("text**

**mining"). Was this type of interaction turned off for this analysis? Please the setting used (and rationale).**

We used all channels of the STRING database, because they are all vetted sources of information about functional protein-protein interactions. The details of how each of the 7 channels in STRING are generated and their rationales are in the manuscript describing STRING, which is cited in our paper.

We believe that the reviewer's concern – that "interactions" between genes based on text mining are generated anytime the gene names appear together in the literature – stems from a misunderstanding of how text-mining interactions are generated in STRING.

The text-mining channel of the STRING database implements a biomedical specific large language model (LLM) called RoBERTa (Lewis et al "Pretrained language models for biomedical and clinical tasks: understanding and extending the state-of-the-art" *Proceedings of the 3rd Clinical Natural Language Processing Workshop* 2020) whose performance on relation extraction tasks has been independently evaluated (Miranda et al "Overview of drugprot biocreative VII track: quality evaluation and large scale text mining of drug-gene/protein relations" *BioCreative* 2021) and that has been trained and evaluated on >240,000 manually annotated relations.

To address this confusion, we have changed the methods to make it clear that we used all channels:

Line 791,
"Interactions from the STRING database were retrieved from https://string-db.org for *Bacillus subtilis* strain 168 (taxid: 224308). The composite channel, which integrates information from all channels (neighborhood, fusion, co-occurrence, co-expression, experiments, database, and text mining) from the STRING database, was used."

We have not changed the main text, since the sentence on line 174 (previously line 172) already reads:

Line 174,
"We found that gene pairs with high absolute GI scores (both positive and negative) were enriched in all interactions documented in STRING (Figure 2B). "

**9. Figure 2D. I do not think there is enough information for readers to find the raw data used, for example, in Figure 2D. Which set of RF data form the basis of this heatmap? What transfers are compared? And why were these pairs chosen? This is generally true for similar figures -- please specify the actual source file for the raw data used to generate the plots!**

All the data discussed in the main text is from fitness after 10 generations of growth, which is consistent with our previous work (Hawkins et al *Cell Systems* 2020). To clarify this ambiguity, we have made the following changes:

Line 140,
"Cells were sampled immediately before dCas9 induction and after 10 doublings post induction, as in our previous work[26]. Several other timepoints were also collected, sequenced, and analyzed (Figure S2, Table S2-S4). The relative fitness (RF) of each strain was calculated by comparing its relative abundance at the start and end of each experiment[26,30,31] (Methods; Table S2)."

We have added a sentence to the Figure 2 legend:

"Data from Table S3 (0-10 generations)."

**10. Figure 3C. As above. The basis for this heatmap (in the extensive SI datasets) is not obvious. It would be very helpful for readers to understand how you used the raw data to make these graphs.**

See our response to point #9.

**11. line 182. Why unexpected? Based on the schematic given and prior work, I would say that these are expected interactions. Perhaps "previously unreported" would be more appropriate.**

We have changed the text as the reviewer suggested.

Line 183,
"as well as previously unreported negative GIs between hydrolases and PG synthases"

**12. Line 186. Why a novel interaction? The mreB and mbl genes are well known to genetically interact.**

We thank the reviewer for pointing out the ambiguity in our writing. The sentence was not meant to refer to an interaction between *mreB* and *mbl*, but rather to interactions involving either *mreB* or *mbl* with other genes. We changed the sentence so it now reads:

Line 187,
"These novel interactions identify new partners for well-studied genes such as the essential actin homologs *mreB* and *mbl* (Figure S5C), as well as for less studied genes, indicating that our data set can function as an engine for discovery (Table S3)."

**13. Section after line 191. A supplemental table of correlated GI profiles would be useful! For example, of the large set of poorly characterized genes, readers may want to see if their favorite gene has a correlated profile with other genes. This work has apparently been done, so why not include the results as a new SI table?**

We apologize for the confusion; the correlations were already provided in Table S4. To clarify this important point, we have added a reference to that table in the introduction of the section:

Line 196:
"Consistent with this idea and with analyses in yeast[37] and human cells[14], we found that gene pairs with highly correlated GI profiles were enriched in previously discovered interactions (Pearson's $r > 0.5$, ~7.3-fold enriched, 141/300 interactions in STRING; Figure 3B and Table S4)."

**14. Line 220. I do not think the word "paralogous" is justified here. Some of the mentioned gene pairs are paralogs, but I think others are not.**

We thank the reviewer for pointing this out. Although *mreB*/*mbl* and *ltaS*/*yfnI* are paralogs, *uppP*/*bcrC* are not. We have made edits to reflect this point:

Line 218,
"**GI analysis reveals distinct functions of paralogous or isofunctional gene pairs**
Duplication and divergence of genes are major drivers of evolution, and as a result, paralogous genes are common in bacteria[42], as are isofunctional (but non-homologous) genes. However, our understanding of the shared and distinct functions of such pairs is often incomplete. We examined the GI profiles of 3 paralogous or isofunctional gene pairs to assess their degree of functional divergence: the non-homologous undecaprenyl pyrophosphate phosphatases *bcrC* and *uppP* (Figure 4A), the paralogous lipoteichoic acid (LTA) synthases *ltaS* and *yfnI* (Figure 4B), and the actin homologs *mreB* and *mbl* (Figure S5C).
*bcrC* and *uppP* each encode an undecaprenyl pyrophosphate phosphatase, a key enzyme in the lipid II cycle and have approximately equivalent transcript levels[43]."

**15. Line 286. YdaH has been renamed Amj. The use of current gene names is encouraged, but since all the raw data has BSU# for each gene this is not a major barrier to further analysis.**

Thank you, we have now changed "*ydaH*" to "*amj*".

**16. I got lost in Fig. 5. There are candidate suppressors in Fig. 5A, but apparently**

**none of these suppress? A bit more explanation would help to tie the description (in results) to the figure.**

Thank you for pointing this out. We updated the figure but neglected to update the legend. We have now done so, as described below:

**Figure 5. GIs disentangle the roles of *mbl* and *mreB* in cell envelope homeostasis.** Data from Table S2 and S3 (0-10 generations).
**A)** *mbl* suppressors for follow-up are indicated in the blue quadrant. Suppressors chosen have an RF > 0.9 and a GI score > 3 relative to the mean RF~0.78 ( gray dot) of *mbl*-control strains in *mbl* double-CRISPRi strains.
**B)** *mbl* suppressors were involved in various aspects of WTA, LTA, and PG synthesis. *glmR* is a positive regulator of UDP-GlcNAC synthesis. UDP-GlcNAC is a precursor for PG and other metabolites.
**C)** Viability of Δ*mbl* or Δ*mreB* in *mbl*-specific and common suppressor backgrounds. *mbl::kan* or *mreB::kan* fragments were transformed into each strain and visualized after 16 hr (Methods). Top: Representative images of the transformation plates of □*ptsI*, □*yfnI*, and □*glmR* strains. Bottom: Summary of viability of all double mutants, with viable pairs indicated by yellow squares.
**D)** Viability of individually constructed double-CRISPRi knockdown strains of all *mbl*/suppressor pairs. Viable knockdown pairs are indicated as in Figure 5C.
**E)** Comparison of cell width changes obtained from microscopy in two groups of strains: set 1, the double knockdown set (CRISPRi knockdown of each *mbl* suppressor + knockdown of *mbl)*; Set 2: the knockdown/deletion set (*mbl* knockdown + suppressor deletion). In CRISPRi OFF strains (black dots), Mbl is present, and suppressors are either present (Set 1) or deleted (Set 2). In CRISPRi ON strains (red dots), Mbl levels are highly reduced and suppressor levels are either highly reduced (Set 1) or absent (Set 2). Note that the *mbl* only strain has a single sgRNA targeting *mbl*. Although all measurements have error bars, in general, the error bars for Set 1 (vertical) are much smaller than those for set 2 (horizontal).
**F)** Comparison of cell growth measurements obtained from bulk cultures, calculated as area under the growth curve (AUC) using strain sets and CRISPRi induction as in Figure 5E.
**G)** Quantitative comparison of the extent of rescue of cell morphology and cell growth using strains and data in Figures 5E and 5F.

**17. Line 674. What it "t10" in this equation?**

We have changed the sentence describing the equation to read:

Line 733,
"where $r_x(t_i)$ is the fraction of strain $x$ in the population after $i$ doublings and $g_{WT}$ is the number of generations of wild-type growth in the experiment."

**18. Line 702. Define MAD.**

We added a definition of MAD:

Line 759
"“Control” columns were those with column medians within 1 median absolute distance (MAD, a robust analog of standard deviation) of the median of column medians, and “control” rows were those with row medians within 1 MAD of the median of row medians.”

**19. Line 719. Not clear what "one-hot" means,**

We modified the text to clarify one-hot encoding. Moreover, we added a reference to our previous manuscript (Hawkins et al *Cell Systems* 2020), which also made use of one-hot encoding. The text now reads:

Line 777,
“For each sgRNA in position 1 (rows), we performed a linear regression between its GI scores and the first 2 nucleotides of the interacting sgRNA. The first 2 nucleotides were one-hot encoded to convert these categorical variables into a matrix of binary values that can be used in linear regression, as previously described[26]”

**20. Line 38: Not exclusively….Much of classical genetics was based on altered function mutations (e.g. temperature sensitive, etc.) in addition to loss of function.**

We agree that many temperature-sensitive (ts) mutants have been analyzed. However, ts mutants are overwhelmingly conditional loss-of-function mutations, often because the altered protein is not stable at high temperature. The text now reads:

Line 39,
“The field of genetics has been built on deducing gene functions by associating mutations with phenotypes.”

**21. Please check grammar in media 1.**

We have checked this text and made minor grammatical changes.

**22. The SI tables are potentially useful, but the intent of the different comparisons (in the different tabs) is not obvious. Please add text to explain, and make sure the cells do not have errors in them.**

We have modified the supplemental tables to clarify their contents. The interpretation for

all pairwise comparisons is described in the “preface” sheet in the Table S2, S3, and S4 in addition to Table S7

**Reviewer #2: Overall comments：**
**Understanding the genetic interactions (GIs) between functional genes can help us decipher their important biological significance. The biological traits reflected by some important genes are still not deciphered, and even these genes are closely related to important research fields such as environmental control and antibiotic resistance.**
**In this manuscript, the author developed double-CRISPRi technology for genome-scale GI screen of envelope genes in model Gram-positive bacterium Bacillus subtilis. The idea of selecting the envelope process is very nice (as it is closely related to pathogenicity and drug resistance), and the analysis of this process can deepen our understanding of the envelope process of Gram-positive bacteria cells and provide a new paradigm for Gram-positive bacteria. The author used this technology to identify over 1,000 known and novel positive and negative GIs. They also demonstrated that paralogous genes are not functionally similar or redundant, but rather play different roles such as the mreB and mbl actin homologs. In summary, the selected research subjects for this study are excellent, the manuscript is well written, and the results are reliable. However, several issues need to be clarified before this study can be accepted.**

**1. The expression of dCas9 is induced through the xylose-induced promoter system in this double-CRISPRi system. As far as I know, xylose promoter has certain leakage expression in B. subtilis. Does this affect the conclusion of the experiment?**

The xylose promoter does indeed have some leakage (see our previous manuscript Peters et al., *Cell* 2016). However, this leakage is not relevant to the interpretation or the conclusions of our experiment. The abundance of all sgRNAs is measured <u>immediately before</u> adding inducer and after 10 generations of growth with inducer. Therefore, if some sgRNAs were depleted due to leaky expression of dCas9, this would affect the starting abundance but not the fold change in abundance during the experiment. We note that we have used this CRISPRi system and analysis strategy in previous publications and found that we were able to accurately quantify the fitness of essential genes (Hawkins et al., 2020).

**2. I agree with the author on the fact that repeat sequences are more likely to be lost in B. subtilis because B. subtilis has a rich DNA recombination system. However, repeat sequences (such as sgRNA scaffold) are more prone to recombination and deletion when DNA bends, as designed by the author.**

We appreciate the reviewer's consensus about the issues that can be caused by recombination.

**3. Figure 5C: ∆mreB ∆ptsI and ∆mbl ∆ptsI showed significant differences in colony size, suggesting that mreB and mbl appear to be equally important for cell**

**growth (perhaps of different importance or with other genes acting as complements). However, the ΔmreB ΔyfnI mutant showed significant growth inhibition compared to the Δmbl ΔyfnI mutant, where mreB and mbl seemed to play different roles. Perhaps mreB and mbl play different roles under specific conditions (But it's not absolute.), thus, I do not agree that mreB and mbl are "distinct roles" as described in the Abstract.**

We completely agree with the reviewer's point that *mreB* and *mbl* have both shared and distinct roles. As such, we have changed the sentence at issue in the abstract so it now reads:

"Our analysis pipeline and experimental follow-ups reveal the shared and distinct roles of paralogous genes such as *mreB* and *mbl* in peptidoglycan and teichoic acid synthesis and identify new genes involved in the well-studied process of cell division."

**4. Figure 6C: Using the constructed double mutants, the author found that all four y-genes exhibited negative GIs with ezrA but not gpsB. The author seem to have missed the negative control ΔgpsB ΔezrA.**

It is not clear to us why the reviewer considers Δ*gpsB* Δ*ezrA* a negative control. GpsB and EzrA are known to interact and constitute a synthetic sick pair, likely due to their combined effects on PBP1 (Claessen et al., 2008). To avoid any confusion, we edited the text to explicitly mention the phenotypes of the Δ*gpsB* Δ*ezrA* strain:

Line 336,
"EzrA also works with GpsB to recruit PBP1 to the division septum[67] and activate PrkC[68]. Our dataset verified the previous finding that *ezrA* and *gpsB* are synthetic sick[67]"

Moreover, as we discuss in the manuscript, it is very likely (and supported by the literature) that some of these new genes function through GpsB.

From the manuscript:
Line 340,
"Despite strong negative GIs with *ezrA*, neither *yrrS*, *ytxG,* or *yerH* exhibited strong negative GIs with these known EzrA partners, raising the possibility that these uncharacterized genes function with one of the EzrA partners. Consistent with this hypothesis, YrrS in *B. subtilis* and YtxG in *S. aureus* have been reported to physically interact with GpsB[71, 72]."

**5. Single-cell imaging (Figure 7B): Lack of control (amyE::Kan), please add.**

This control has now been added.

**Minor issues:**
**1. Figure 6C: Please use the subscript below for OD600**

We made the change as suggested.

**2. Figure 6C: Please change "∆yterH" to "∆yerH"**

We made the change as suggested.

**3. Summary: line 26: but it is difficult to implement at the genome-scale.**

We made the change as suggested.

**4. Summary: line 28: We discover > 1000 known to "We discover > 1,000 known"**

We made the change as suggested.

**5. Introduction: line 40: high throughput techniques to "high-throughput techniques"**

We made the change as suggested.

**6. Introduction: line 82: identification of synthetic-lethal gene pairs to "the identification of synthetic-lethal gene pairs"**

We made the change as suggested.

**7. Introduction: line 86: "envelope-function genes necessary to" to "envelope-function genes is necessary to"**

We changed the sentence.
Line 83, “envelope-function genes, which is necessary to”

**8. Results: line 105: Because loss of repeated to "Because the loss of repeated"**

We made the change as suggested.

**9. Results: line 118: select essential genes to "selected essential genes"**

We made the change as suggested.

**10. Results: line 185: data set to "dataset"**

We made the change as suggested.

**11. Results: line 210: highly correlated to that of genes in their regulon to "highly correlated with those of genes in their regulon"**

We made the change as suggested.

**12. Results: line 242: Other GIs with bcrC motivate additional study to "Other GIs with bcrC motivate additional studies"**

We made the change as suggested.

**13. Perspective: line 432: gene function and pathway connections to "gene functions and pathway connections"**

We made the change as suggested.

**14. Perspective: line 459: mis-localization to "mislocalization"**

We made the change as suggested.

**15. Limitations: line 512: yrrS unaffected by pbpI knockdown to "yrrS that is unaffected by pbpI knockdown"**

We made the change as suggested.

**Reviewer #3: The manuscript by Koo & Todor et al deals with the construction and characterization of double-CRISPRi libraries in Bacillus subtilis, with a specific focus on cell envelope genes. On the whole I believe the work presented in the manuscript will be a useful resource for those studying the cell envelope in related bacteria. A strength of the manuscript is that the double-CRISPRi approach was able to recapitulate existing genetic interactions and reveal new ones. Generally speaking however, I found that the manuscripts runs a little short on the functional characterization of the most interesting, novel genetic interactions, particularly if we consider the plethora of cell biology tools available to characterize the bacterial cell envelope. As a systems biology focused manuscript, it does however fulfil the purpose of elucidating existing and novel genetic interactions and providing a novel resource for the community of cell envelope enthusiasts.**

**Specific comments:**

**Knockdowns - can the authors present evidence of knockdown looking specifically at the transcript levels for the genes that they chose to focus on in for example in Figure 6 & 7?**

The CRISPRi system we use was established in Peters et al. *Cell* 2016 (and has been used in many publications since). In that manuscript, we used both RNA-seq and flow cytometry to show that, when induced, the CRISPRi system results in ~100- to 150-fold knockdown of the targeted gene. To clarify this point, we have edited our initial description of the CRISPRi system to mention the fold-knockdown.

Line 98,
"Our double-CRISPRi system is based on a xylose-inducible, chromosomally-integrated, single-gene knockdown CRISPRi system that effects ~100-fold knockdown of targeted genes[8, 28]."

**Line 139/140 - How did the authors decide on 10 doubling times post-induction?**

Generally, longer experiments can discern weak (near wild-type) phenotypes, while shorter experiments can more accurately measure strong phenotypes. We previously found (Hawkins et al., 2020) that 10 generations is appropriate for the screening of growth phenotypes and confirmed this point in this screen; we also showed that phenotypes after 15 generations were similar to after 10 generations. Note that the GIs at all relevant timepoints are presented in the Supplementary material, for further perusal. We have edited the text to reflect this point as follows:

Line 140,
"Cells were sampled immediately before dCas9 induction and after 10 doublings post induction, as in our previous work[26]. Several other timepoints were also collected, sequenced, and analyzed (Figure S2, Table S2-S4). The relative fitness (RF) of each

strain was calculated by comparing its relative abundance at the start and end of each experiment[26,30,31] (Methods; Table S2)."

**Figure 6C - growth curves - it seems like the authors only conducted two biological replicates. Why not 3?**

The two biological replicates of double-deletions were consistent with each other and with the double CRISPRi data. As such, we did not feel that a third biological replicate was necessary.

**Would it make more sense to present the doubling time instead?**

We think presenting the growth curves is more informative because it allows the reader to ascertain how the mutations influence lag time, maximum growth rate, average growth rate, and maximum optical density - information that would be lost if we just presented the doubling time.

**Figure 7: Why did the authors choose to use phase contrast and then FM4-64 to measure cell length? For the analysis, how did the authors deal with the cells that are visibly stressed? Where these included in the analysis? How many cells were counted? How many biological replicates?**

We used phase contrast to capture cell morphologies and observed elongated cells in the mutants shown in Figure 7. Because phase contrast alone cannot distinguish filamentous cells from chains of cells separated by septa, we stained the cytoplasmic membrane with FM4-64. As shown in Figure 7B, the elongated mutant cells were mostly separated into shorter individual compartments.

In the analysis, we included all cells, both visibly stressed and not. Thus, each statistics in Figure 7A reflects the entire population. We counted $n>200$ cells for each distribution. All images were acquired in a single microscopy session, which captured the major phenotypes (cell elongation and cytoplasmic compartmentalization) with enough cells to represent the distribution of cellular dimensions. We have updated the figure caption to include these details.

**Figure 7: Did the authors also measure the interchromosome (i.e. internucleoid) distance? It seems like several of these mutants (ytxG, yerH) may have a larger interchromosomosal distance? Did the authors also determine the frequency of cell lysis?**

We appreciate the reviewer's interesting observation. We agree that the interchromosome distance appears larger in *ezrA-KD* Δ*ytxG* and *ezrA-KD* Δ*yerH* cells compared to wild type. Currently, the underlying causes and implications of these

physiological differences are unclear. We chose to not include an analysis of this distance in the current manuscript, as a mechanistic explanation would require a systematic investigation into the growth behavior and nucleoid organization for each mutant that is beyond the scope of this work. However, this point could be interesting motivation for a follow-up study on nucleoid dynamics and spatial organization.

In Figure 7D, we measured the frequency of lysis in FM4-64-stained cells exhibiting bright foci, focusing on the relationship between envelope defects and lysis in these mutants.

**Figure S2 - legend - can the authors include more detail in the legend - it would be useful to have the text between line 645 and 658 as the legend for this figure.**

We have updated and expanded the Figure S2 legend to better explain the figure. It now reads:

"**Figure S2**. Related to Figure 1. **Schematic of the pooled growth experiment and sampling time points.** Straight blue arrows represent growth without xylose (CRISPRi system OFF). Straight red arrows represent growth with 1% xylose (CRISPRi system ON). Broken lines represent back dilution. $OD_{600}$ are denoted below each flask. Green arrows represent timepoints at which cells were collected (T0-T7). Samples were collected at these time points to determine the optimal generation times for the GI screen during exponential growth and to assess GI during recovery from overnight culture. The interpretation for all pairwise comparisons is described in the "preface" sheet in the Table S2, S3, and S4 in addition to Table S7.  For each timepoint, replicates were obtained from the cultures of three independent 1liter flasks."

**Responses to the reviewers' comments**

**Manuscript Number: CELL-SYSTEMS-D-24-00429R1**
**"Comprehensive genetic interaction analysis of the Bacillus subtilis envelope using double-CRISPRi"**

*Note: Reviewer comments are in bold, author responses are in regular font.*

**Reviewers' comments:**

**Reviewer #2: The authors have addressed most of my previous concerns and improved the manuscript accordingly. The revisions are satisfactory, and the paper now meets the journal's standards.**

We are grateful for Reviewer #2's constructive feedback throughout the review process. We are glad to hear that the revisions have satisfactorily addressed the concerns.

**Reviewer #3: The authors have addressed most of my comments. However, the comments about the data in Figure 7A have not been addressed.**

**The authors state: In the analysis, we included all cells, both visibly stressed and not. Thus, each statistics in Figure 7A reflects the entire population. We counted n>200 cells for each distribution. All images were acquired in a single microscopy session, which captured the major phenotypes (cell elongation and cytoplasmic compartmentalization) with enough cells to represent the distribution of cellular dimensions. We have updated the figure caption to include these details.**

**However, the figure caption has not been updated and this data is not from 3 biological replicates but from a single experiment. How do the authors know these data are representative? It seems like these experiments were done only once and not repeated. Furthermore, the authors include statistics, but they have not mentioned what statistical test was used. This needs to be addressed because it is hard to know if this data is reproducible or not. Furthermore, it is not clear what statistical test was used. The authors need to be demonstrate reproducibility in their work.**

We thank the reviewer for this comment and have updated the figure legend to clarify that the data shown are from a single imaging session involving one biological replicate. We have also ensured that all statistical information is included in the figure legends, including the specific tests used and the number of cells (See below).

We acknowledge that additional biological replicates would further support reproducibility. However, we note that the main point of this figure—specifically, that the apparent elongation in the left plot of Fig. 7A is not representative of significant elongation in individual cells—is clearly substantiated by the FM4-64 labeling data. As such, this panel serves as a supporting observation rather than a central result of the study. Given the minor nature of this point and the consistency with other data in the paper, particularly the NeonGreen-FtsZ data that we focused on acquiring to extend our conclusions for the revision, we believe that repeating this specific imaging experiment is not essential and would unnecessarily delay the overall dissemination of our findings.

We hope the clarifications and revisions now sufficiently address the reviewer's concerns.

*Updated figure legend (all statistical information is shown in blue)*

Figure 7. Phenotypes of knockout mutants of several uncharacterized genes in combination with *ezrA* depletion.

**A)** Depletion of EzrA causes apparent filamentation in Δ*gpsB* and several previously uncharacterized mutants (Δ*yerH*, Δ*ypbE*, Δ*yrrS*, and Δ*ytxG*) compared to *ezrA* knockdown alone or gene knockouts without *ezrA* knockdown, as indicated by phase-contrast microscopy (left). However, the length of cell compartments delineated by FM4-64-labeled membrane staining showed limited increase compared to the control strain, suggesting unperturbed Z-ring localization (right). All images were acquired in a single imaging session involving one biological replicate. $n > 200$ cells were quantified for each distribution. Statistical significance was calculated using Welch's t-test. **: $p < 0.01$; ***: $p < 0.001$.

**B)** The elongated mutant cells caused by *ezrA* knockdown are separated into shorter compartments based on FM4-64-labeled division planes. In all mutants, cell division planes occurred away from the DAPI-labeled nucleoid, as normally seen in wild type. Scale bar: 5 μm.

**C)** *ezrA* knockdown alone or together with Δ*gpsB*, Δ*yrrS*, Δ*ypbE*, Δ*ytxG*, or Δ*yerH* increased the fraction of Z-rings with prolonged lifespan, leading to persistent Z-rings at the adjacent new poles after cell division in all mutants except *ezrA*-KD/Δ*gpsB*. $n \geq 17$ Z-rings were tracked for each strain. Statistical significance was calculated using Welch's t-test.

**D)** The fraction of cells containing bright foci and those developing shape defects or undergoing lysis is dependent on the deletion of specific genes and knockdown of *ezrA*, underscoring the impact of genetic interactions on cell division defects. The fraction of cells with bright foci increased in double mutants regardless of induction of *ezrA* knockdown, likely because basal knockdown of *ezrA* combined with each gene deletion is sufficient to increase this phenotype. $n > 50$ cells were counted for each combination of strain and condition, except for *ezrA* Δ*gpsB* with induction of the CRISPRi system ($n = 19$) due to relatively low cell viability.
